# Physiotherapists’ perception of barriers and facilitators to the implementation of digital health interventions: a scoping review

**DOI:** 10.1186/s12913-026-14836-0

**Published:** 2026-06-04

**Authors:** Evelina Baniunaite, Luis Antonio Stängl, Eveline S. Graf, Laura Wittich

**Affiliations:** 1 Charité, Universitätsmedizin Berlin, Berlin, Germany; 2 ZHAW Zurich University of Applied Sciences, Winterthur, Switzerland; 3 Department of Health Care Management, Institute for Technology and Management, Faculty VII - Economics and Management, Berlin University of Technology, Berlin, Germany; 4 Department of Health Care Management, Technical University of Berlin, Berlin, Germany

**Keywords:** Digital health interventions, Physiotherapy, Implementation, CFIR, Health workforce

## Abstract

**Background:**

Digital health interventions (DHIs) hold significant potential to enhance physiotherapy by improving patient engagement, enabling remote care, and optimizing treatment outcomes. Despite their benefits, the slow pace of implementation indicates barriers to the adoption of DHIs. Together with the limited research on physiotherapists’ perspectives, this highlights the need to address barriers and facilitators to their integration. Understanding these factors is crucial for advancing the effective use of DHIs in physiotherapy practice.

**Objective:**

This scoping review aimed to identify and map barriers and facilitators to the implementation of DHIs in physiotherapy, as perceived by physiotherapists.

**Methods:**

The JBI methodological framework and the JBI reviewer’s manual guided the review process. MEDLINE Ovid and Embase Ovid were the primary databases, with BASE used for grey literature; the search process was completed in May 2024. Barriers and facilitators were mapped to the updated Consolidated Framework for Implementation Research (CFIR).

**Results:**

The review included 45 publications spanning 2017 to 2024. A total of 334 barrier instances and 134 facilitator instances were identified across all five CFIR domains. Key factors affecting the implementation of DHIs in physiotherapy were highlighted, including the need for adaptable, well-designed, and compliant interventions, as well as supportive reimbursement systems and adequate funding. In addition, successful DHI integration in clinical practice is influenced by reliable technology, technical training, institutional investment, and an open attitude toward technology.

**Conclusion:**

The findings indicate that successful DHI integration within physiotherapy services may require coordinated action across organizational, workforce, and policy domains. Health system strategies that address infrastructure capacity, digital competencies, and reimbursement models appear critical to translating digital innovation into routine service delivery, though further research examining objective implementation outcomes is warranted.

**Registration:**

OSF (https://osf.io/abhxr)

**Supplementary Information:**

The online version contains supplementary material available at 10.1186/s12913-026-14836-0.

## Background

The development and deployment of digital technologies are becoming increasingly vital in the healthcare sector [1]. The COVID-19 pandemic served as a catalyst, accelerating the integration of digital technologies into healthcare practices in response to the outbreak [2–4]. The application of these technologies to address challenges within healthcare systems, support healthcare workforces, and optimize individual health outcomes is referred to as digital health interventions (DHIs) [5, 6].

The potential of DHIs is also evident in the field of physiotherapy [7, 8]. Telehealth and mHealth can expand access to care and achieve outcomes comparable to traditional approaches [9–13]. These interventions may support personalized care and enhance patient engagement; however, their implementation in routine practice remains variable and context-dependent [14–17]. Due to the breadth of these technologies, DHIs comprise diverse, discrete functionalities. In line with the World Health Organization (WHO) [6], a DHI is defined as “a discrete functionality of digital technology that is applied to achieve health objectives”. Thus, DHIs denote the intervention-level use of digital technologies in care, whereas “digital health” describes the broader field and “digital technologies” the underlying tools and platforms.

Many countries have made considerable progress in digitizing their healthcare systems [4], bolstered by initiatives such as the introduction of reimbursable digital health applications [4, 14]. While DHIs are increasingly integrated into physicians’ practices, their adoption in physiotherapy remains relatively limited [18–20]. Understanding healthcare professionals’ perspectives is essential for identifying implementation factors and enhancing the acceptance and uptake of digital technologies [21]. However, existing research in this field has predominantly focused on physicians and nurses, leaving a gap in understanding physiotherapists’ views on DHI implementation [18, 22].

To date, no systematic effort has comprehensively explored physiotherapists’ perspectives on the barriers and facilitators to implementing DHIs across diverse clinical settings and intervention types. Existing scoping reviews addressing physiotherapists’ perceptions have been narrowly focused, such as on barriers related specifically to video-call-based telerehabilitation [23], or general telerehabilitation [24]. Consequently, despite the growing use of DHIs, there is limited synthesis of physiotherapists’ perspectives on barriers and facilitators to their implementation across diverse contexts.

Given the heterogeneity of DHIs in physiotherapy across intervention modalities, clinical contexts, and reported outcomes, a scoping review was deemed appropriate to map the available evidence on implementation determinants as perceived by physiotherapists. The review aimed to identify and characterize barriers and facilitators influencing the implementation of DHIs, as perceived by physiotherapists.

## Methods

### Overview

The scoping review was conducted in accordance with the methodological framework recommended by the Joanna Briggs Institute (JBI) for scoping review [25] and guided by the JBI Reviewer’s Manual [26]. The reporting process adhered to the PRISMA Extension for Scoping Reviews (PRISMA-ScR) guidelines [27]. A prospectively registered protocol, developed in accordance with the PRISMA-P statement [28], was uploaded to the Open Science Framework in May 2024, and updated in July 2024 under: https://osf.io/abhxr.

### Eligibility criteria

The eligibility criteria were guided by the Population, Concept, Context (PCC) framework [26], as detailed in Supplementary Table S1. Articles were included if they assessed the implementation of DHIs in physiotherapy and reported on perceived barriers, facilitators, or both from the perspective of physiotherapists across various physiotherapy settings. These settings included all potential locations for the provision of physiotherapy services, such as rehabilitation facilities, outpatient clinics, inpatient units, private practices, and specialized environments for specific conditions. Implementation was defined as the adoption or use of DHIs, whether in the past, present, or planned for the future. Barriers were identified as factors impeding the adoption or use of DHIs, while facilitators were those that enabled their integration.

As the WHO’s definition of DHIs [6] encompasses a broad range of technologies, including telehealth systems enabling remote service delivery, mHealth applications, electronic health records, wearable devices, and emerging technologies such as AI applications, “digital health” is referred to as the overarching field, “digital technologies” as the underlying tools and platforms, and “DHIs” as the specific application of these technologies to achieve defined health outcomes in physiotherapy practice.

The inclusive approach to DHI types was adopted because implementation determinants at the practice level often transcend specific technology categories, and factors such as workflow integration, training needs, reimbursement, and attitudes toward technology affect adoption across modalities. This broad scope aligns with the exploratory purpose of scoping reviews while acknowledging that subtype-specific determinants may also exist.

Studies relevant to the research question but lacking a clear distinction between populations were excluded. All study types were considered, except for study protocols, conference abstracts, and research posters, which were excluded due to incomplete information. No language restrictions were applied, and non-English or non-German sources were translated using a web-based translator (*DeepL SE*, Cologne, Germany) in 2024. Only articles accessible in full-text format were eligible. To ensure applicability to contemporary DHI development and implementation contexts—given the rapid evolution of DHIs, infrastructure, and regulatory requirements—only studies published between 2014 and 2024 were included.

### Search strategy

The search strategy was developed in collaboration with experienced librarians. Primary electronic databases, including MEDLINE Ovid and Embase Ovid, were utilized, along with BASE to identify grey literature. The search process was completed in May 2024.

The search terms included synonyms and MeSH terms related to digital health interventions, implementation, and physical therapy. Facilitators and barriers were not used as search terms due to sensitivity reasons, as they are often not explicitly mentioned in the title or abstract. The search strategy was developed for MEDLINE Ovid, and then adapted to all remaining databases (see Supplementary Table S2). The search strategy was validated by ensuring that the key publications identified in advance were included in the search results. As an additional source of information, reference lists of included studies were searched using the Citation Chaser web application [29]. Furthermore, a hand search was conducted in Google Scholar using key terms on DHIs as defined in the search strategy, combined with the term “physiotherapy,” to capture literature not retrieved through the database searches. The first five pages of results were screened for relevance. Expert recommendations were also considered to identify further relevant studies.

### Study screening and selection

All identified studies were first transferred to the citation management program EndNote 21. Subsequently, duplicates were removed using the “Deduplicator” tool available on the Systematic Review Accelerator (SRA) website [30]. SRA tool “Screenatron” was used for the screening process. A pilot test was conducted using a random sample of 25 titles and abstracts, screened independently by EB and LS. Initial agreement exceeded 75%. Disagreements were infrequent and resolved through discussion, with a third reviewer (LW) involved where necessary, resulting in full consensus. Full-text versions of potentially relevant sources were then retrieved and screened against the eligibility criteria by EB and LS. LW reviewed all excluded articles to ensure that no pertinent information was overlooked.

### Data extraction

The extraction form was pilot-tested and refined after data were extracted from five publications. Variables included authors, year, country, type of DHI, sample size, physiotherapists’ sex and age, study context, facilitators, barriers, and data type (qualitative or quantitative). Facilitators and barriers were extracted from the results sections of the included studies, regardless of whether they were presented as explicit determinants or as broader themes, provided they could be clearly mapped to the pre-specified CFIR coding guideline. Data were extracted at the level of authors’ reported findings (including interpreted themes), rather than from individual participant quotations, unless a quotation was explicitly presented as a barrier or facilitator. The study context covered factors such as delivery conditions, specialization, health issues, or care settings. EB independently extracted data using Microsoft Forms, preserving the original text, and transferred it to Microsoft Excel, where LS verified accuracy.

### Data synthesis

Extracted data were summarized descriptively and presented using narrative summaries, tables, and graphs. Percentages were rounded to one decimal place, and data visualization was performed in RStudio (R version 4.0.2) and Pandas 2.2.3 for Python 3.11.

DHIs were classified into the following categories:

- Telehealth Systems: This category included telerehabilitation, telehealth, and other forms of digital remote service delivery.
- mHealth: This category included mobile-based services.
- Integrated Digital Health Management Systems: This category included tools such as electronic billing or scheduling.
- Blended interventions combined face-to-face and digital components.
- Other DHIs were classified by purpose, technological modality, or, when unclear, based on definitions in the publications.

Data on barriers and facilitators were analyzed and mapped onto the updated Consolidated Framework for Implementation Research (CFIR) [31]. The CFIR is a systematic, meta-theoretical model designed to identify and evaluate barriers and facilitators to the implementation of interventions [32]. The CFIR comprises 67 constructs, providing a structured approach to mapping and understanding implementation determinants. The constructs are categorized into five domains:

1. **Innovation** refers to the specific characteristics of the intervention, including its complexity and adaptability.
2. **Outer Setting** encompasses external influences such as policies, regulations, and local conditions that may impact implementation.
3. **Inner Setting** focuses on the organizational environment where the innovation is introduced, considering factors like available resources and structural dynamics.
4. **Individuals** highlight the people involved in the process, taking into account their knowledge, attitudes, and beliefs.
5. **The Implementation Process** addresses strategies, planning, and activities that drive the successful adoption of the intervention.

To ensure consistency in mapping each barrier and facilitator, a coding guideline was developed based on the updated CFIR (see Supplementary Table S3). The framework recommends defining and operationalizing constructs for the project, including creating additional constructs to cover all relevant topics [31]. In this review, the Individuals domain was defined to include characteristics of both physiotherapists and patients, as perceived from the physiotherapists’ perspective. The Inner Setting was defined as the environment where physiotherapy is delivered, including clinical institutional settings, and patients’ homes. To capture all relevant themes, the construct “Attitude toward technology/DHIs” was introduced during coding and added to the Individuals domain. This construct was intended to reflect individual-level acceptance, trust, and scepticism toward digital health interventions, which were not fully captured by the existing constructs of the updated CFIR framework. The CFIR coding was conducted by one reviewer (EB) and verified against the coding guideline by a second author (LS), with discrepancies resolved through discussion. Because coding was not performed independently by two coders, formal inter-coder agreement statistics were not assessed.

## Results

### Selection of sources of evidence

**Fig. 1:**
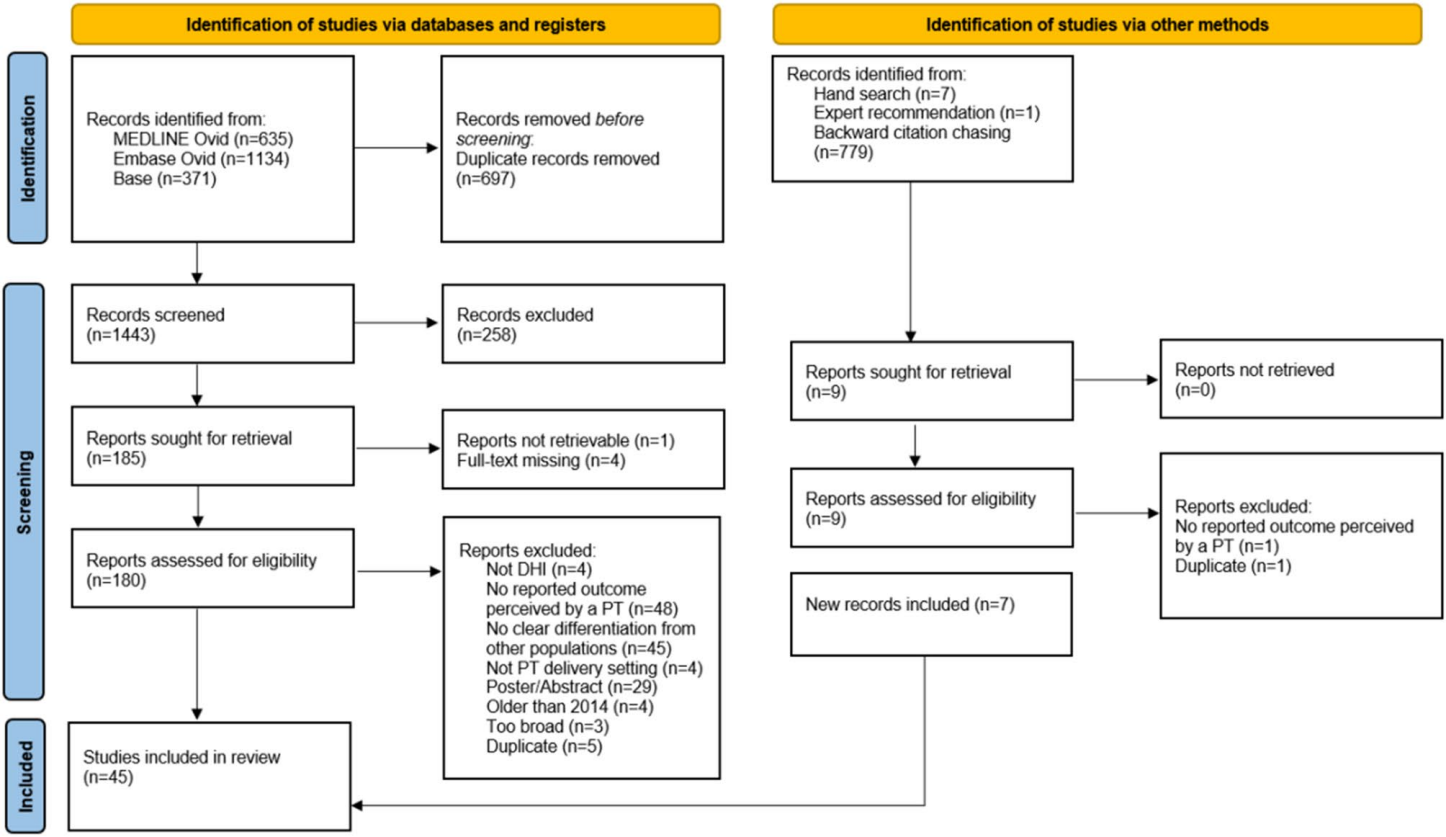
The PRISMA flowchart. Legend: The PRISMA flowchart shows the individual steps of the study identification process

A systematic database search conducted in May 2024 identified 2,140 studies (MEDLINE Ovid: *n* = 635; Embase Ovid: *n* = 1,134; BASE: *n* = 371). Following the removal of duplicates (*n* = 697), 1,443 articles underwent title and abstract screening, yielding 185 for retrieval. Of these, four full-texts were unavailable, and one could not be retrieved, leaving 180 articles for full-text screening. Based on the eligibility criteria, 142 articles were excluded, as detailed in the PRISMA flowchart [33] (see Fig. 1). A list of excluded studies with reasons is provided in Supplementary Table S4.

An additional 787 articles were identified through alternative methods, including hand searches (*n* = 7), expert recommendations (*n* = 1), and backward citation chasing (*n* = 779). After title and abstract screening, 778 articles were excluded, and nine were retrieved. Of these, seven met the inclusion criteria. In total, 45 studies were included in the scoping review. No included studies were published in languages other than English or German.

### Study characteristics

Of the 45 included studies, the majority (91.1%) were journal articles reporting primary research, while 6.7% were Master’s theses and 2.2% consisted of a scoping review. The studies were published between 2017 and 2024, with most appearing from 2020 onward. In terms of methodologies, 44.4% employed qualitative approaches, 31.1% used quantitative methods, and 24.4% adopted mixed methods (see Table 1).

**Table 1:** Characteristics of the included studies

Authors	Year	Country of origin^a^	Type of DHI	Type of data collected^b^	PT sample size, sex, age^b^	Context^c^
Allegue et al. [34]	2022	Canada	Telehealth systems (combined with exergames)	Qualitative	n = 2	Stroke^e^
Aloyuni et al. [35]	2020	Saudi Arabia	Telehealth systems	Quantitative	n = 347 (*n* = 70 female; *n* = 106 male)	Governmental and private hospitals
Alsobhi et al. [36]	2022	N/A	AI applications	Qualitative and quantitative	n = 236 (38.8% female; 59.6% male)	Rehabilitation^e87^
Arzani et al. [37]	2022	Iran	Telehealth systems	Quantitative	n = 192 (50% female; 50% male) Mean age: 37.38 years, SD (±10.5)	N/A^e^
Augustine et al. [38]	2024	India	Telehealth systems	Quantitative	n = 201	Developing country^e^
Bennell et al. [39]	2021	Australia	Telehealth systems	Qualitative and quantitative	n = 207 (73% female; 27% male)	N/A^e^
Bezuidenhout et al. [40]	2022	Sweden	Telehealth systems	Quantitative	Neurology *n* = 139 (93% female) <30 years (4%); ≥60 years (9%) Geriatrics *n* = 168 (89% female) <30 years (5%); ≥60 years (9%)	Neurology; Older adults^e^
Bhattarai et al. [41]	2020	Australia	Self-management support (mHealth)	Qualitative	n = 8	Older adults; Arthritis
Björklund O. [42]	2022	Finland	Telehealth systems	Qualitative	n = 11	Rehabilitation^e^
Braakhuis et al. [43]	2021	Netherlands	Wearables	Qualitative and quantitative	n = 103 (*n* = 76 female; *n* = 26 male) Mean age: 42.2 years, SD (±12.06)	Stroke^e^
Button et al. [44]	2018	UK	Self-management support (web-based)	Qualitative	n = 6	Knee injuries
Colón-Semenza et al. [45]	2023	USA	Telehealth systems	Qualitative	n = 5	Parkinson’s disease^e^
Cottrell et al. [46]	2017	Australia	Telehealth systems	Qualitative	n = 15 (*n* = 6 female; *n* = 9 male)	Neurosurgical; Orthopedic
Dissanayaka et al. [47]	2022	Sri Lanka	Telehealth systems	Quantitative	n = 268 (58.6% female; 41.4% male)	Knee osteoarthritis^e^
Dyck K. [48]	2019	Canada	Integrated Digital Health Management Systems	Quantitative	n = 11 (80.7% female; 19.3% male) Mean age: 46.2 years	Various
Ede et al. [49]	2024	Nigeria	Home treatment programs (Not limited to a specific technology modality)	Qualitative	n = 12 (*n* = 7 male)	Older adults^e^
Erturan et al. [50]	2024	Türkiye	Telehealth systems	Quantitative	n = 219 (59% female; 41.1% male)	Developing country^e^
Ezzat et al. [51]	2023	Canada	Telehealth systems	Qualitative	n = 1409	N/A^e^
Guggenberger et al. [52]	2022	Austria	Telehealth systems	Qualitative	n = 8 (50% female; 50% male)	Outpatient setting^e^
Hague et al. [53]	2024	Canada	Telehealth systems	Quantitative	n = 274	Outpatient and community settings^e^
Haines et al. [54]	2023	Australia	Telehealth systems	Qualitative	n = 19 (89% female)	Outpatient setting^e^
Hawley-Hague et al. [55]	2023	UK	Telehealth systems	Qualitative and quantitative	Qualitative: *n* = 12 Quantitative: *n* = 1620	N/A^e^
Heiskanen et al. [56]	2021	Finland	Telehealth systems	Qualitative and quantitative	n = 250	N/A^e^
Hellstén et al. [57]	2023	Finland	Telehealth systems	Quantitative	n = 579; (83.2% female; 16.8% male) Mean age: 48.8 years SD (±11.9)	N/A^e^
Keel et al. [58]	2023	Switzerland	Digital exercise prescription and monitoring (mHealth)	Qualitative	n = 13	Outpatient setting^e^
Kelly et al. [59]	2022	Ireland	Self-management support (Not limited to a specific technology modality)	Qualitative	n = 13; (*n* = 6 female; *n* = 7 male) Mean age: 35 years Age range: 26–42	Musculoskeletal disorders^e^
Kloek et al. [60]	2018	Netherlands	Blended intervention (Web-based)	Qualitative and quantitative	Quantitative: *n* = 49 Qualitative: *n* = 9 (*n* = 3 female; *n* = 6 male) Age range: 24–59 (median 52 years)	Osteoarthritis
Lord Ferguson S. [61]	2024	Canada	Telehealth systems	Qualitative and quantitative	n = 30 (50% female)	Private practice^e^
Marschütz J. [62]	2022	Austria	Telehealth systems	Qualitative	n = 12 (*n* = 8 female; *n* = 4 male) Age range: 24–38 years	Freelance; Orthopedic^e^
Marwaa et al. [63]	2020	Denmark	Patient-centered treatment (mHealth applications, ICT)	Qualitative	n = 4	Stroke
McDermott and Bradley [64]	2023	Ireland	Health condition management (Not limited to a specific technology modality)	Quantitative	n = 139	Parkinson’s disease^e^
Miller et al. [65]	2022	USA	Telehealth systems	Qualitative	n = 13, (69% female; 31% male)	N/A^e^
Pelckmans et al. [66]	2023	Netherlands	eHealth interventions^**d**^ (Not limited to a specific technology modality)	Qualitative and quantitative	Quantitative *n* = 154 (92.8% female) Mean age: 49.1 years, SD (±10.8) Qualitative *n* = 16 (93.8% female) Mean age: 44.3 years SD (±10.0)	Pediatric^e^
Rausch et al. [67]	2021	Switzerland	Telehealth systems	Quantitative	n = 742 (74.1% female, 23.5% male, 1.7% missing; 0.7% unknown) Mean age: 43 years; SD (±11)	N/A^e^
Rettinger and Kuhn [23]	2023	Various	Telehealth systems (Video call-based)	Qualitative and quantitative	n = 2857	Various
Reynolds et al. [68]	2021	Ireland	Telehealth systems	Qualitative and quantitative	n = 205 (77.56% female; 22.44% male) Mean minimum age: 36 years Mean maximum age: 45 years	N/A^e^
Roitenberg and Ben-Ami [69]	2022	Israel	Telehealth systems	Qualitative	n = 17 (*n* = 11 female, *n* = 6 male) Mean age: 43 years	N/A^e^
Ross et al. [70]	2023	Australia	Telehealth systems	Qualitative	n = 25 (68% female; 32% male) Mean age 36.8, SD (±11.2)	Public hospital^e^
Rowe and Sauls [71]	2020	South Africa	mHealth applications	Quantitative	n = 270 (92% female) Age range: 20–70 years	N/A
Saaei and Klappa [72]	2021	USA	Telehealth systems	Qualitative and quantitative	n = 228 (72% female, 26% male; 2% preferring not to answer) Age range 25–70 years (50% 25–40 years)	Various^e^
Sia et al. [73]	2024	Malaysia	Telehealth systems	Qualitative	n = 24 (75% female, 25% male) Age range: 30–39 years (>50%)	Musculoskeletal disorders^e^
Slevin et al. [74]	2020	Ireland	Health condition management (Not limited to a specific technology modality)	Qualitative	n = 4	COPD
Suzer and Buker [75]	2023	Türkiye	Telehealth systems	Quantitative	n = 237 (67.09% female, 32.91% male) Mean Age: 33.37 years, SD (±9.36)	Various^e^
van der Meer et al. [76]	2020	Netherlands	Health condition management (Not limited to a specific technology modality)	Qualitative	n = 11 (63.6% female) Mean age: 43.1 years Age range: 28–63 years	Temporomandibular disorders^e^
Vasanthi et al. [77]	2021	Malaysia	Digital physiotherapy intervention (Not limited to a specific technology modality)	Quantitative	n = 209 (59.30% female, 40.70% male) Mean age: 29.93 years, SD (±4.69)	Various^e^

a: Refers to the country where the data was collected

b: Refers to the outcome of interest, gender distribution and age added, if specified

c: Refers to the study’s focus, including conditions under which DHIs are delivered, physiotherapy specialization, specific health issues, or the care setting

d: Refers to the definition used in the publication, including “provider-centric electronic record, patient-centric electronic record, health information exchange, and telehealth”

e: Refers to data collected during or after the outbreak of the COVID-19 pandemic

Various – diverse contexts (more than three described)

AI – artificial intelligence

ICT – information and communication technology

mHealth – mobile-based health services

PT – physiotherapist

SD – standard deviation

N/A – no answer; physiotherapy not further specified

Legend: The table provides an overview of studies on DHIs in physiotherapy, including authors, publication years, countries, types of interventions, data collected, and study contexts

The included studies were conducted across 20 countries, with one scoping review covering multiple nations and one study not specifying its country of origin. European countries contributed the largest share (42.2%), followed by North America (17.8%), Asia (15.6%), Australia (11.1%), and African countries and Türkiye, as a transcontinental nation, each contributing 4.4%.

The studies covered a range of physiotherapy settings; however, in 4.4% of studies, the setting was not specified. Telehealth systems were the most frequently studied digital health intervention, appearing in 62.2% of studies. Notably, 77.8% of studies collected data during or after the COVID-19 pandemic.

### Barriers and facilitators

**Fig. 2:**
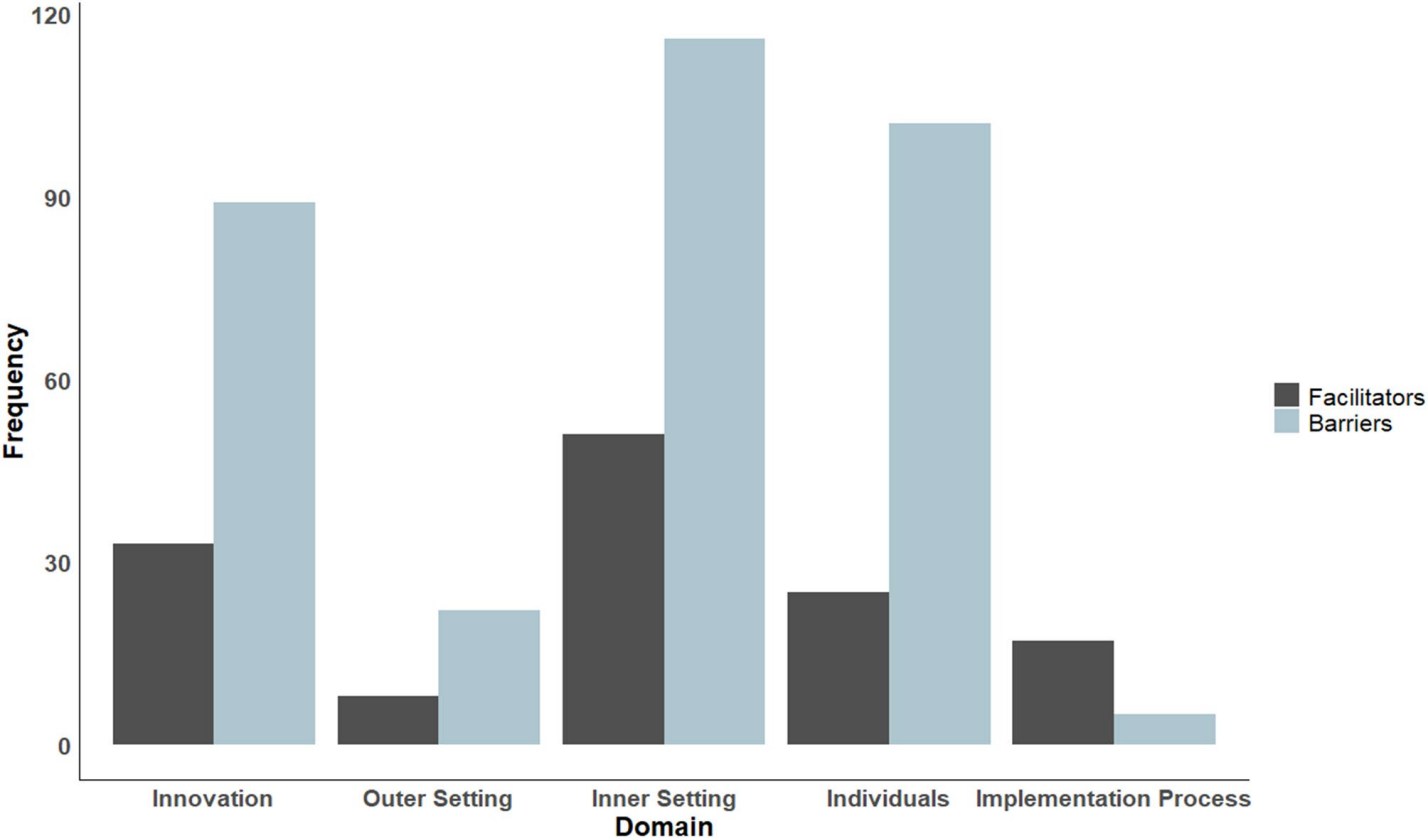
Facilitators and barriers to DHI implementation across the CFIR domains. Legend: This bar chart represents the frequency of facilitators and barriers across different domains. The x-axis displays five domains: innovation, Outer Setting, Inner Setting, Individuals, and implementation process. The y-axis indicates the frequency of these factors

A total of 334 barriers and 134 facilitators to the implementation of DHIs across all CFIR domains were identified (see Fig. 2).

**Fig. 3:**
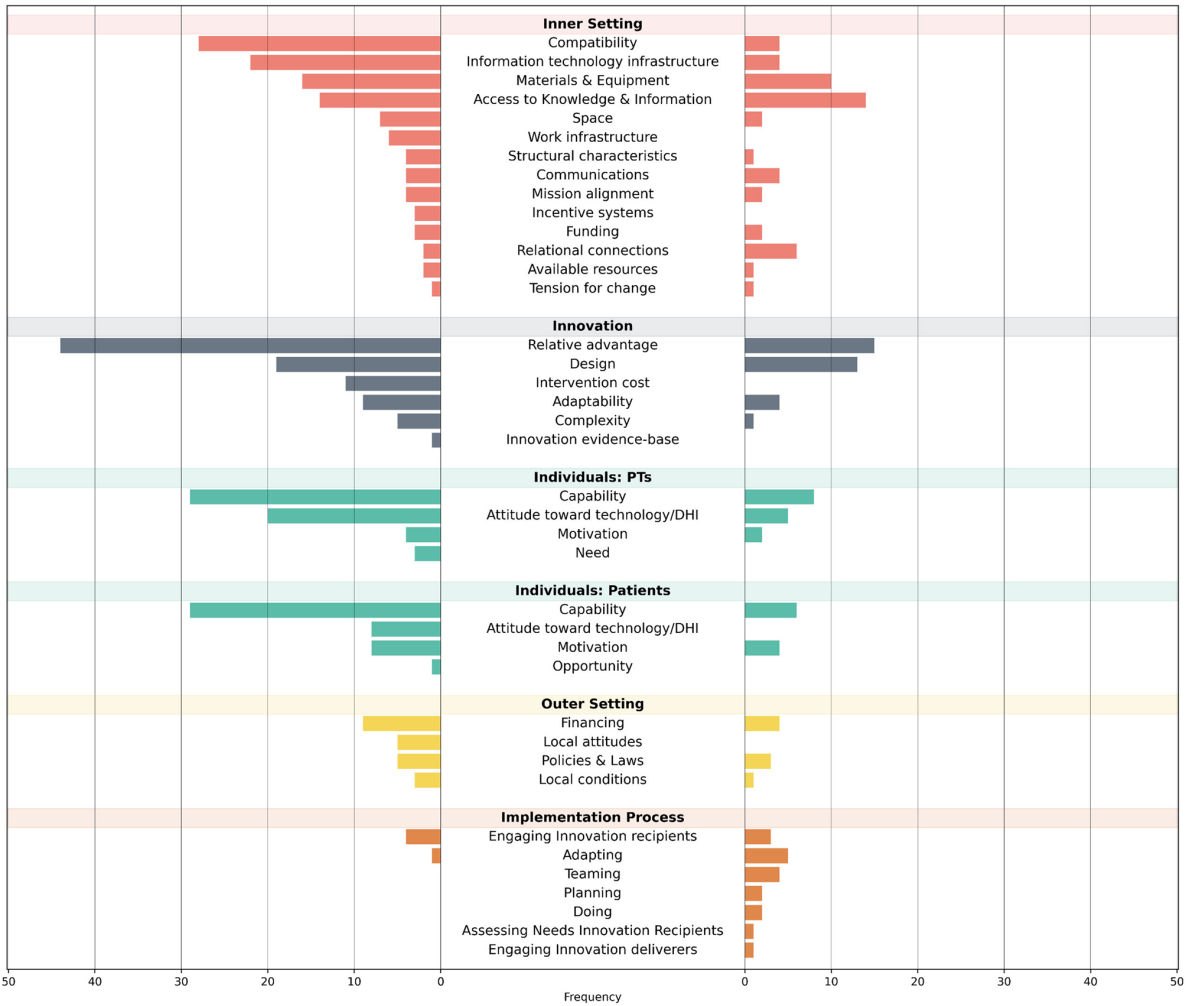
Barriers and facilitators across the CFIR constructs. Legend: This chart illustrates the frequency of barriers (left) and facilitators (right) across constructs within the CFIR implementation domains, with the x-axis indicating frequency and the y-axis grouping constructs by domain

Barriers were most commonly identified in the Inner Setting (*n* = 116, 34.7%), followed by Individuals (*n* = 102, 30.5%) and Innovation (*n* = 89, 26.6%), whereas the Outer Setting (*n* = 22, 6.6%) and the Implementation Process (*n* = 5, 1.5%) were least frequently cited. Additionally, four unspecified barriers could not be assigned to CFIR constructs (e.g., labeled as “others” or “don’t know”). Similarly, the Inner Setting had the most facilitators (*n* = 51, 38.1%), followed by Innovation (*n* = 33, 24.6%) and Individuals (*n* = 25, 18.7%), with fewer in the Implementation Process (*n* = 17, 13.7%) and Outer Setting (*n* = 8, 6.0%), as shown in Fig. 3.

The **Inner Setting** domain accounted for the highest frequency of barriers and facilitators. Most barriers were observed in the *Compatibility* construct (*n* = 28), including increased workload and insufficient time for consultations [44]. Similarly, facilitators (*n* = 4) emphasized aspects such as the interoperability of DHIs with existing tools [58]. In *Access to Knowledge & Information*, barriers (*n* = 14) included inadequate training and education for physiotherapists [52], while facilitators (*n* = 14) included educational programs, technical support [57], and experienced mentoring [70], which improved DHI adoption.

Barriers in *Information Technology Infrastructure* (*n* = 22) included poor connectivity and technical issues [35, 58], whereas stable internet connections facilitated adoption (*n* = 4) [37]. In the *Materials & Equipment* construct, barriers (*n* = 16) included lack of access to essential devices such as smartphones [38], while facilitators (*n* = 10) involved access to necessary technology for both physiotherapists [53] and patients [65]. In *Space*, barriers (*n* = 7), such as the absence of dedicated telehealth rooms, hindered implementation [68], whereas their availability facilitated their implementation (*n* = 2).

The *Communications* construct identified barriers (*n* = 4), including limited collaboration with information and communication technology (ICT) specialists [50], while facilitators (*n* = 4) involved clear communication between therapists and patients [38]. Under *Relational Connections*, barriers (*n* = 2) arose when family involvement altered communication dynamics [56], whereas facilitators (*n* = 6) included pre-existing physiotherapist-client relationships [39] and family members assisting with the DHI [62].

Barriers in *Work Infrastructure* (*n* = 6) included a lack of skilled personnel and poor task management [46, 75], with no reported facilitators. In *Mission Alignment*, insufficient leadership and lack of institutional support were barriers (*n* = 4) [51], whereas strong senior leadership served as a facilitator [54] (*n* = 2). Barriers under *Structural Characteristics* (*n* = 4) included preferences for non-compliant devices that conflicted with organizational security policies, whereas institutional guidelines facilitated implementation [55] (*n* = 1).

In *Funding*, barriers (*n* = 3) included a lack of financial resources for acquiring necessary equipment [65], whereas institutional financial support (*n* = 2) acted as a facilitator [57]. *Incentive Systems* were constrained (*n* = 3) by concerns that telerehabilitation could lead to job insecurity for physiotherapists [50], with no facilitators reported. Barriers in *Available Resources* (*n* = 2) included insufficient time to explore alternative solutions [63], whereas allocated work hours (*n* = 1) supported implementation [57]. *Tension for Change* was hindered (*n* = 1) by resistance to change [46], whereas a perceived need for change acted as a facilitator [46] (*n* = 1).

The **Innovation** domain also made notable contributions. Within *Relative Advantage*, barriers (*n* = 44) included the perceived need for face-to-face interactions [50] and potential depersonalization of care [68]. Facilitators (*n* = 15) included reduced patient travel time, improved healthcare access [66], increased exercise adherence [51], and reduced infection risks during COVID-19 [60]. In *Design*, barriers (*n* = 19) involved lack of user-friendly software [50], privacy concerns [48], and patient safety risks for high-risk individuals [55]. Facilitators (*n* = 13) highlighted intuitive app designs and patient safety assurance [34, 52].

In *Adaptability*, barriers (*n* = 9) included a lack of individualized programs [49] and challenges in managing specific conditions [36], whereas facilitators (*n* = 4) emphasized DHIs’ ability to address individual healthcare needs [76]. The *Cost* construct reported only barriers (*n* = 11), with high costs as a key challenge [35]. Under *Complexity*, barriers (*n* = 5) included complex interfaces and usability issues [72], while simplicity of use facilitated (*n* = 1) adoption [38]. The *Evidence-Base* construct identified the lack of published standards as a barrier (*n* = 1) to implementation [38].

The **Individuals** domain highlighted barriers and facilitators among physiotherapists and patients. In *Capability*, barriers for physiotherapists (*n* = 29) included insufficient knowledge about DHIs [36], while facilitators (*n* = 8) included confidence in technology [57] and greater work experience [47]. For patients, barriers (*n* = 29) included older age, reduced dexterity, and limited technological literacy [41], whereas facilitators (*n* = 6) included younger age, prior familiarity with technology, and satisfaction with DHIs [45]. Within *Motivation*, barriers among physiotherapists (*n* = 4) included low willingness [35], while among patients (*n* = 8), they included lack of readiness [58]. Facilitators for physiotherapists (*n* = 2) included enthusiastic leadership [55], while for patients (*n* = 4), willingness to adopt DHIs facilitated implementation [39]. In *Attitudes Toward Technology/DHI*, barriers among physiotherapists (*n* = 20) and patients (*n* = 8) included negative attitudes and preconceptions [23], whereas trust and acceptance of technology facilitated adoption (*n* = 5) for physiotherapists [46]. Lastly, in *Opportunity*, barriers (*n* = 1) included patients’ inability to access technology due to cultural or religious factors [69]. The *Need* construct suggested that physiotherapists might perceive DHIs as unnecessary when alternative methods were sufficient, thereby posing a barrier (*n* = 3) [68].

The **Outer Setting** domain contributed fewer barriers and facilitators. In *Financing*, barriers (*n* = 9) included the lack of reimbursement for telerehabilitation services [52], whereas reimbursement availability acted as a facilitator (*n* = 4) [69]. In *Policies & Laws*, barriers (*n* = 5) included unclear frameworks regarding certification, liability, and data privacy [52], whereas facilitators (*n* = 3) included well-defined mHealth regulations [52]. In *Local Attitudes*, barriers (*n* = 5) included providers exclusively requesting inpatient sessions [65], with no facilitators reported. In *Local Conditions*, barriers (*n* = 3) included limited national financial resources [49], while offering face-to-face therapy alongside digital interventions facilitated adoption (*n* = 1) [35].

The **Implementation Process** domain had the least representation. In *Engaging Innovation Recipients*, barriers (*n* = 4) included difficulties motivating patients [56], whereas facilitators (*n* = 3) involved fostering a supportive environment [38]. In *Adapting*, barriers (*n* = 1) included difficulties accommodating diverse staff and patient needs [60], whereas facilitators (*n* = 5) involved tailoring resources and approaches. In *Teaming*, barriers (*n* = 4) included low adaptive capacity [39, 55, 60], whereas collaboration and resource sharing acted as facilitators (*n* = 4) [51]. In *Engaging Innovation Deliverers*, peer encouragement among colleagues served as a facilitator (*n* = 1) [54]. In *Planning*, facilitators (*n* = 2) included preparing resources and providing clear instructions [39], while hands-on learning experiences in the *Doing* construct further supported implementation [54].

## Discussion

This scoping review identified barrier and facilitator instances to implementing DHIs in physiotherapy and mapped them across all CFIR domains, drawing on 45 publications from diverse countries and physiotherapy settings.

The findings highlight the multifaceted nature of DHI implementation. We were able to assign both facilitators and barriers to a range of constructs, such as Compatibility, Information Technology Infrastructure, and Capability, suggesting that these may be foundational to successful implementation. For instance, a stable internet connection is fundamental, as its absence directly creates a barrier. In contrast, constructs identified solely as facilitators, such as *Planning*-related factors (e.g., providing clear instructions and offering hands-on learning experiences), may reflect desirable but non-critical enhancements to the implementation process. These distinctions highlight the importance of distinguishing between essential requirements and supplementary elements when strategizing DHI implementation, at least from the physiotherapists’ perspective [78].

Most studies collected data during or after the COVID-19 pandemic, underscoring its role in accelerating digital technology adoption [2, 79]. However, this exceptional context must be considered when interpreting the findings, as it likely reshaped priorities and dynamics. The urgency of digital adoption during the pandemic may have led to a stronger focus on immediate, internal factors rather than broader, systemic influences.

Accordingly, barriers and facilitators were primarily identified within the *Inner Setting*, *Individuals*, and *Innovation* domains, emphasizing immediate influences such as individual perceptions, institutional contexts, and DHI characteristics. However, the lower representation of determinants in the Outer Setting and Implementation Process domains should not be interpreted as evidence of lesser importance. Rather, several factors may explain this pattern. Many of the included primary studies were qualitative and may not have been guided by implementation frameworks, potentially limiting their attention to broader system-level determinants. In addition, because these studies focused largely on physiotherapists’ perspectives, participants may have been more likely to comment on visible, practice-level issues—such as workflow, usability, and everyday operational challenges—than on less visible external factors, including financing mechanisms, legal frameworks, or policy structures that lie outside their direct experience, but which are recognized as crucial for DHI adoption [9, 80, 81]. The rapid implementation of digital tools during the pandemic, often in the absence of well-defined legal or reimbursement mechanisms, may further explain the limited focus on these external factors in the reviewed studies [82, 83].

The **Inner Setting**, encompassing the characteristics of the environment where an innovation is implemented, emerged as the most frequently cited domain for both barriers and facilitators. This underscores the pivotal role of internal organizational dynamics in determining implementation success. Key barriers included increased workload and insufficient time for consultations, with physiotherapists often experiencing stress due to demanding schedules [84–86]. Similarly, Rothgangel et al. [85] identified an imbalance between workload and perceived benefits as a challenge in adopting telemonitoring platforms. These findings highlight the need to integrate DHIs into existing workflows to prevent overburdening physiotherapists. Access to appropriate technology, platform functionality, and a stable internet connection also emerged as critical factors. Technological and connectivity challenges were particularly pronounced in developing countries [38, 50], consistent with findings from Barton et al. [87], who examined patient perceptions of remote physiotherapy. In contrast, Ross et al. [88] reported minimal technical issues in videoconferencing for knee osteoarthritis, suggesting that financial and infrastructural disparities may contribute to differing experiences across settings. Additionally, training and experience in both physiotherapy and digital technologies, captured in the *Individuals* domain, also shape perceptions of technological barriers [89].

Barriers within the **Innovation Domain** included the perceived need for face-to-face therapy and concerns about a weakened therapist-patient relationship. These challenges align with previous research indicating that healthcare professionals, including physiotherapists, view a diminished therapist-patient relationship as a key limitation of DHIs [84, 90]. Resistance to departing from traditional care models further restricts DHI adoption, despite its potential to enhance patient outcomes and streamline workflows.

The perceived advantages and design of DHIs significantly influenced implementation. When DHI design aligns with clinical needs, it facilitates adoption; however, misalignment creates barriers. A major contributing factor is the limited involvement of physiotherapists in DHI development, as they often prefer tools designed by their peers over those produced by other providers [18]. These findings underscore the need for greater collaboration during the development process to ensure DHIs are tailored to physiotherapy practice.

In the **Individuals Domain**, negative attitudes and limited capability among both physiotherapists and patients were prominent barriers. These attitudes influence perceptions of the relative advantages of DHIs, often shaped by individual beliefs and technological literacy. Incorporating digital training and education on the benefits of DHI into physiotherapy curricula, as recommended in recent research [91, 92], could help mitigate these challenges and foster greater confidence in technology.

The relevance of the **Implementation Process** has been emphasized in the literature, particularly regarding the need for collaboration among diverse stakeholders, including public authorities, ICT providers, physiotherapy associations, patient groups, insurers, and regulators [89]. A coordinated approach may streamline implementation and ensure alignment across sectors, ultimately improving outcomes.

Importantly, the identified barriers are not limited to operational implementation challenges but also raise broader equity and ethical concerns. Limited access to devices, poor connectivity, low digital literacy, privacy concerns, and variations in reimbursement are not merely technical obstacles, but raise critical questions about fair access, inclusion, patient autonomy, and the potential for digital exclusion.

A recent rapid review examining ethical and equity dimensions in telerehabilitation have demonstrated that disparities related to age, socioeconomic status, geography, gender, and ethnicity can systematically disadvantage certain patient groups [93]. Older adults, in particular, may face compounded barriers including reduced digital literacy, limited access to devices, and conditions affecting dexterity—barriers prominently identified in this review. The concentration of patient-related capability barriers (*n* = 29) in our findings, including older age and limited technological literacy, aligns with broader concerns about digital exclusion. Furthermore, privacy concerns identified as barriers in our review reflect patients’ legitimate concerns about autonomy and data protection. As DHIs become more prevalent, ensuring equitable access and addressing ethical dimensions of digital care delivery must be considered alongside technical implementation factors. Policy and practice responses should explicitly address these equity implications rather than treating implementation as a purely operational challenge.

Ultimately, implementation determinants may vary by DHI subtype. While this review adopted a broad, inclusive approach appropriate for scoping the evidence landscape, specific technology categories—such as synchronous telehealth versus asynchronous mHealth applications, or AI-based systems versus administrative platforms—may present distinct implementation challenges related to their differing workflows, risk profiles, and infrastructure requirements. Future research examining subtype-specific determinants would complement these findings.

## Strengths and limitations

This scoping review’s strength lies in its comprehensive approach, which addresses a research gap by incorporating a broad range of study designs, diverse physiotherapy settings, and various DHIs. The inclusion of studies in all languages ensured a thorough and inclusive examination of the available evidence.

However, certain limitations should be noted. The literature search was last conducted in May 2024. Consequently, studies published thereafter were not captured, and the findings should be interpreted as reflecting the evidence available up to that time. Despite exhaustive efforts, the full text of one potentially relevant publication could not be obtained, introducing a slight risk of selection bias.

Furthermore, the assessment of facilitators and barriers did not account for variation in the degree of agreement across studies. Within individual studies, some factors were supported by only a few participants, whereas others had broader consensus. Overall, the identified determinants should be interpreted as perceived barriers and facilitators reported by physiotherapists, rather than as objective evidence of implementation success.

Another limitation relates to the absence of universally accepted definitions of DHIs, reflecting the field’s rapid evolution. In addition, the review did not differentiate between specific physiotherapy settings or subfields, which may affect applicability across different contexts. Certain groups may exhibit greater resistance or differing responses depending on their practice environment.

Furthermore, CFIR constructs are often interconnected, meaning the frequency of specific constructs should be interpreted cautiously, with greater emphasis placed on overarching domains. Additionally, the introduction of an additional construct into the CFIR framework may limit direct comparability with other studies applying the framework without modification.

Lastly, the review did not critically appraise the quality of the included studies, consistent with the purpose of scoping reviews, which aim to provide an overview of evidence rather than assess methodological rigor [94].

## Conclusion

This review mapped perceived barriers and facilitators to DHI implementation in physiotherapy and highlights several determinants that may be relevant to adoption and integration. Across studies, characteristics of DHIs (e.g., perceived adaptability, usability, safety considerations, and privacy/compliance requirements) were frequently described as shaping implementation. Reimbursement arrangements, financial resource availability, and reliable technology and connectivity were also reported as important contextual conditions. Additionally, institutional support, such as provision of technical training, dedicated spaces, and administrative resources, was commonly perceived as facilitating integration. Limited time and workload pressures, as well as digital skills, motivation, and attitudes toward technology among physiotherapists and patients, were repeatedly described as potential enablers or barriers.

Future research should prioritize the involvement of physiotherapists in DHI development to ensure that tools align with clinical needs and workflows, thereby enhancing usability and adoption. Investigating effective training methods for integrating DHIs into practice and improving digital competencies among both practitioners and patients is essential. Moreover, further research should explore the impact of DHIs on the patient-therapist relationship, addressing concerns about maintaining therapeutic connections in digital contexts.

Finally, robust evidence on the effectiveness and cost-effectiveness of DHIs is needed to justify their integration into routine physiotherapy care and to ensure their sustainable adoption. Demonstrating their value in improving patient outcomes will be key to fostering long-term implementation and acceptance.

## Electronic supplementary material

Below is the link to the electronic supplementary material.

Supplementary material 1

## Data Availability

Data generated or analyzed during this study are included in this published article and its supplementary information files.

## Abbreviations

AI: Artificial Intelligence
BASE: Bielefeld Academic Search Engine
CFIR: Consolidated Framework for Implementation Research
COPD: Chronic Obstructive Pulmonary Disease
DHIs: Digital Health Interventions
ICT: Information and Communication Technology
JBI: Joanna Briggs Institute
mHealth: Mobile Health
ML: Machine Learning
N/A: Not Applicable/Not Available
PRISMA: Preferred Reporting Items for Systematic Reviews and Meta-Analyses
PRISMA-ScR: Preferred Reporting Items for Systematic Reviews and Meta-Analyses Extension for Scoping Reviews
PRISMA-P: Preferred Reporting Items for Systematic Review and Meta-Analysis Protocols
PT: Physiotherapist/Physical Therapist
SRA: Systematic Review Accelerator
VR: Virtual Reality
WHO: World Health Organization
