# Supplementary material for: Physiotherapists’ perception of barriers and facilitators to the implementation of digital health interventions: a scoping review
Source: BMC Health Serv Res. 2026 Jun 4;26:794. doi: 10.1186/s12913-026-14836-0 (PMC13242126; doi:10.1186/s12913-026-14836-0)
Supplement: Supplementary file 1 — Supplementary material 1 [file 12913_2026_14836_MOESM1_ESM.pdf]

SUPPLEMENTARY MATERIAL
Manuscript title: Physiotherapists' Perception of Barriers and Facilitators to the Implementation of Digital Health Interventions: A Scoping Review

Authors: Evelina Baniunaite[1], Luis Antonio Stängl[1], Prof. Dr. Eveline S. Graf[2], Laura Wittich[C,3]
C Corresponding Author
1 Charité – Universitätsmedizin Berlin
2 Institute of Physiotherapy, School of Health Sciences, ZHAW Zurich University of Applied Sciences, Winterthur, Switzerland
3 Department of Healthcare Management, Institute for Technology and Management, Faculty VII - Economics and Management, Berlin University of Technology, Berlin, Germany

Table S1 PCC Criteria

Inclusion	Exclusion
**Population**<br>- Physiotherapists	**Population**<br>- Unclear distinction of the findings from other populations
**Concept**<br>- Intervention: DHIs<br>- Primary outcomes: Facilitators and barriers to the implementation of DHIs	**Concept**<br>- Not specified in the characteristics, or broad description<br>- No facilitators or barriers to implementation perceived by a physiotherapist
**Context**<br>- All physiotherapy settings<br>- All languages<br>- Full-text available 2014-2024	**Context**<br>- Study protocols<br>- Conference abstracts or research posters

Table S2 Search strategies

Ovid MEDLINE(R) ALL <1946 to May 24, 2024>

1	Digital Health/	265
2	Telemedicine/	39551
3	Electronic Health Records/	28324
4	Telerehabilitation/	1159
5	Mobile Applications/	12415
6	Digital health intervention*.ti,ab.	1010
7	mHealth.ti,ab.	6200
8	m-health.ti,ab.	638
9	eHealth.ti,ab.	4626
10	e-health.ti,ab.	3155
11	digital health application*.ti,ab.	207
12	digital health.ti,ab.	6210
13	telerehabilitation.ti,ab.	1885
14	tele health.ti,ab.	226
15	mobile application*.ti,ab.	5407
16	web based intervention*.ti,ab.	1517
17	digital tool*.ti,ab.	2013
18	ICT.ti,ab.	8101
19	skype.ti,ab.	549
20	artificial intelligence.ti,ab.	41673
21	artificial intelligence application*.ti,ab.	564
22	wearable*.ti,ab.	28637
23	DiGA.ti,ab.	177
24	digital therapeutic care app*.ti,ab.	2
25	1 or 2 or 3 or 4 or 5 or 6 or 7 or 8 or 9 or 10 or 11 or 12 or 13 or 14 or 15 or 16 or 17 or 18 or 19 or 20 or 21 or 22 or 23 or 24	169690
26	Physical Therapists/	3417
27	Physical Therapy Modalities/	41532
28	physio therap*.ti,ab.	65
29	physiotherap*.ti,ab.	35324
30	physical therap*.ti,ab.	29995
31	26 or 27 or 28 or 29 or 30	87038
32	implement*.ti,ab.	751687
33	adopt*.ti,ab.	364160
34	integrat*.ti,ab.	758535
35	feasibility.ti,ab.	259884
36	innovation*.ti,ab.	82918
37	usage.ti,ab.	132599
38	uptak*.ti,ab.	440153
39	assimilat*.ti,ab.	33765
40	32 or 33 or 34 or 35 or 36 or 37 or 38 or 39	2557108
41	25 and 31 and 40	635

Embase Classic+Embase <1947 to 2024 May 24>

1	Digital Health.ti,ab.	7161
2	telemedicine/	47690
3	electronic health record/	45116
4	mobile application/	23661
5	digital health intervention*.ti,ab.	1011
6	mHealth.ti,ab.	6319
7	m-health.ti,ab.	885
8	eHealth.ti,ab.	5091

9 e-health.ti,ab. 4343
10 digital health application*.ti,ab. 329
11 telerehabilitation/ 3026
12 tele health.ti,ab. 548
13 mobile application*.ti,ab. 7536
14 web based intervention*.ti,ab. 1669
15 digital tool*.ti,ab. 2480
16 ICT.ti,ab. 10842
17 skype.ti,ab. 1042
18 artificial intelligence.ti,ab. 49417
19 artificial intelligence application*.ti,ab. 619
20 wearable*.ti,ab. 31129
21 DiGA.ti,ab. 229
22 digital therapeutic care app*.ti,ab. 2
23 1 or 2 or 3 or 4 or 5 or 6 or 7 or 8 or 9 or 10 or 11 or 12 or 13 or 14 or 15 or 16 or 17 or 18 or 19 or 20 or 21 or 22 220692
24 physiotherapist/ 30587
25 physiotherapy/120556
26 physio therap*.ti,ab. 166
27 physiotherap*.ti,ab. 64359
28 physical therap*.ti,ab. 47194
29 24 or 25 or 26 or 27 or 28 171357
30 implement*.ti,ab. 973980
31 adopt*.ti,ab. 455085
32 integrat*.ti,ab. 914985
33 feasibility.ti,ab. 360567
34 innovation*.ti,ab. 105287
35 usage.ti,ab. 189410
36 uptak*.ti,ab. 580480
37 assimilat*.ti,ab. 34656
38 30 or 31 or 32 or 33 or 34 or 35 or 36 or 37 3269882
39 23 and 29 and 38 1134

BASE
("digital health intervention*" ehealth mhealth "mobile application*" telerehabilitation DiGA) AND (physiotherap* "physical therap*") AND (implement* adopt*)
Hits: 371

Table S3 Coding Guideline for the Consolidated Framework for Implementation Research (CFIR)

Note:

- Individuals were categorized into two groups: physiotherapists and patients (as perceived by physiotherapists)
- The Inner Setting was defined as the environment in which physiotherapy is delivered, encompassing either the institutional setting or the home environment of patients receiving/using DHIs

**I Innovation Domain**	
**Construct Name**	**Definition**<br>*The degree to which:*
Innovation source	The group that developed and/or visibly sponsored use of the DHI is reputable, credible, and/or trustable.
Innovation evidence-base	The DHI has robust evidence supporting its effectiveness.
Innovation relative advantage	The DHI is better than other available innovations or current practice, defined as traditional face-to-face physiotherapy without digital tools. This construct also considers the degree to which hands-on techniques and personal contact are essential in physiotherapy, potentially creating disadvantages with DHI use, such as inaccuracies in information capturing during assessments.
Innovation adaptability	The DHI can be modified to fit the needs of the specific context. It encompasses the extent to which personalized care for specific conditions can be provided, addressing individual needs.
Intervention trialability	The DHI can be tested or piloted on a small scale and undone.
Intervention complexity	The DHI is complicated, which may be reflected by its scope and/or the nature and number of connections and steps.
Intervention design	The DHI is well-designed and user-friendly, offering ease of use, intuitive navigation, and features that enhance usability for all users. This construct also refers to the extent to which the DHI can ensure patient safety, including physical well-being, and the degree to which privacy and data security are maintained.
Intervention cost	The DHI purchase, and operating costs are affordable.
**II Outer Setting**	
**Construct Name**	**Definition**<br>*The degree to which:*
Critical incidents	Large-scale and/or unanticipated events disrupt implementation and/or delivery of the DHIs.
Local attitudes	Sociocultural values encourage the Outer Setting to support implementation and/or delivery of the DHIs. For example, the attitudes and perspectives on DHIs of physicians, who refer patients for physiotherapy.
Local conditions	Economic, environmental, political conditions and geographic location enable the Outer Setting to support implementation and/or delivery of the DHIs.

Partnerships & Connections	The Inner Setting is networked with external entities, including referral networks, academic affiliations, and professional organization networks.
Policies & Laws	Legislation, regulations, professional group guidelines and recommendations, or accreditation standards support implementation and/or delivery of the DHIs.
Financing	Funding from external entities (e.g., grants, reimbursement) is available to implement and/or deliver the DHIs.
External Pressure	External pressures drive implementation and/or delivery of the DHI. For example, regulatory requirements to meet specific standards.
Societal Pressure	Mass media campaigns, advocacy groups, or social movements or protests drive implementation and/or delivery of the DHI.
Market Pressure	Competing with and/or imitating peer entities drives implementation and/or delivery of the DHI.
Performance-Measurement Pressure	Quality or benchmarking metrics or established service goals drive implementation and/or delivery of the DHI.
**III Inner Setting**	
**Construct Name**	**Definition**<br>*The degree to which:*
Structural Characteristics	Institutional/organizational policy guidelines define the use and choice of DHIs within the Inner Setting.
Physical Infrastructure	The layout, configuration of space, and other tangible material features, including the physical environment, all contribute to the functional performance of the Inner Setting.
Information Technology Infrastructure	The information and communication technology (ICT) is operating effectively, encompassing the functionality of hardware, software, and Wi-Fi connectivity.
Work Infrastructure	Organization of tasks and responsibilities within and between individuals and teams, and general staffing levels, support functional performance of the Inner Setting and implementation and/or delivery of DHIs.
Relational Connections	There are high quality formal and informal relationships, networks, and teams within and across Inner Setting boundaries (e.g., structural, professional).
Communications	There are high quality formal and informal information sharing practices within and across Inner Setting boundaries (e.g., structural, professional). It encompasses aspects such as the level of quality communication between physiotherapists and their patients or the patients' relatives, as well as interactions between physiotherapists and professionals from other fields, like IT specialists.
Culture	There are shared values, beliefs, and norms across the Inner Setting.
Human Equality-Centeredness	There are shared values, beliefs, and norms about the inherent equal worth and value of all human beings
Deliverer-Centeredness	There are shared values, beliefs, and norms around caring, supporting, and addressing the needs and welfare of recipients.
Learning-Centeredness	There are shared values, beliefs, and norms around psychological safety, continual improvement, and using data to inform practice

Tension for Change	The current situation is intolerable and needs to change.
Compatibility	The use of DHI fits with workflows, systems, and processes. This construct refers to the extent of workload change through process complexity and interoperability of the DHI among IT systems.<br>This construct also addresses the extent to which the time required to implement and use the DHI fits seamlessly into the workflow, including the session length.
Relative Priority	Implementing and delivering the DHI is important compared to other initiatives.
Incentive Systems	Tangible and/or intangible incentives and rewards and/or disincentives and punishments support implementation and delivery of the DHIs.
Mission Alignment	Implementing and delivering the innovation is in line with the overarching commitment, purpose, or goals in the Inner Setting. Within the context of the delivering institution, it involves the personnel at all levels being aligned and supportive of the implementation process.
Available Resources	Sufficient resources are available to implement and deliver the DHI. This includes the additional time needed to stay updated with current DHIs on the market and explore alternative solutions.
Funding	Funding is available for the implementation and delivery of the DHI.
Space	Physical space is available to implement and deliver the DHI.
Materials & Equipment	Supplies as the appropriate technology or appropriate material for the potential physiotherapeutic exercises are available to implement and deliver the DHI.
Access to Knowledge & Information	Guidance and/or training is accessible to implement and deliver the DHI.
**IV Individuals**	
**Construct Name**	**Definition**<br>*The degree to which:*
Need	The individual(s) has deficits related to survival, well-being, or personal fulfillment, which will be addressed by implementation and/or delivery of the DHI.
Capability	The individual(s) possesses the interpersonal competence, knowledge of potential possibilities of DHIs, and skills required to fulfill the role. This includes physical and cognitive abilities, such as language proficiency, digital literacy, and cognitive functioning.
Opportunity	The individual(s) has availability, scope, and power to fulfill Role. For example, specific communities or groups of individuals might be restricted from using technology.
Motivation	The individual(s) is committed to, willing and compliant with implementing the DHI.
Attitude toward technology/DHIs	The individual(s) accepts, holds favorable beliefs and trusts technology/DHIs.
**V Implementation process domain**	
**Construct Name**	**Definition**<br>*The degree to which individuals:*

Teaming	Join together, intentionally coordinating and collaborating on interdependent tasks, to implement the DHI. This includes sharing knowledge and engaging with colleagues who possess relevant expertise.
Assessing Needs	Collect information about priorities, preferences, and needs of people.
Innovation Deliverers	Collect information about the priorities, preferences, and needs of deliverers to guide implementation and delivery of the DHI.
Innovation Recipients	Collect information about the priorities, preferences, and needs of recipients to guide implementation and delivery of the DHI.
Assessing Context	Collect information to identify and appraise barriers and facilitators to implementation and delivery of the DHI.
Planning	Identify roles and responsibilities, outline specific steps and milestones, and define goals and measures for implementation success in advance. Preparing in advance for sessions and informing patients about what they will need.
Tailoring Strategies	Choose and operationalize implementation strategies to address barriers, leverage facilitators, and fit context.
Engaging	Attract and encourage participation in implementation and/or the innovation.
Innovation Deliverers	Attract and encourage deliverers to serve on the implementation team and/or to deliver the DHI.
Innovation Recipients	Attract and encourage recipients (patients) to participate in the DHI.
Doing	Implement in small steps, tests, or cycles of change to trial and cumulatively optimize delivery of the DHI.
Reflecting & Evaluating	Collect and discuss quantitative and qualitative information about the success of implementation.
Implementation	Collect and discuss quantitative and qualitive information about the success of implementation.
Innovation	Collect and discuss quantitative and qualitative information about the success of the DHI.
Adapting	Modify the innovation and/or the Inner Setting for optimal fit and integration into work processes. Adjusting to available resources and accommodating staff with varying levels of experience.

Damschroder LJ, Reardon CM, Widerquist MAO, Lowery J. The updated Consolidated Framework for Implementation Research based on user feedback. Implementation Science. 2022;17(1).

Table S4 Studies excluded with reasons

**No facilitators/barriers to implementation of DHI perceived by a PT**
• Ahmad, A., Ahlin, K., Mozelius, P., & Hassan Sodhro, A. (2023). Exploring the Medical Caregivers' Perceptions of Technology Acceptance for an Online Speech and Language Assessment Application Among Stroke Patients. http://urn.kb.se/resolve?urn=urn:nbn:se:kau:diva-96096<br>• Alsobhi, M., Khan, F., Chevidikunnan, M. F., Basuodan, R., Shawli, L., & Neamatallah, Z. (2022). Physical Therapists' Knowledge and Attitudes Regarding Artificial Intelligence Applications in Health Care and Rehabilitation: Cross-sectional Study. *Journal of Medical Internet Research*, *24*(10). https://doi.org/https://dx.doi.org/10.2196/39565<br>• Baser Secer, M., & Celiker Tosun, O. (2022). Examination of Telerehabilitation Knowledge, Awareness, and Opinions of Physical Therapy and Rehabilitation Students. *Medical Science Educator*, *32*(6). https://doi.org/https://dx.doi.org/10.1007/s40670-022-01649-z<br>• Blixt, L., Solbrække, K. N., & Bjorbækmo, W. S. (2021). Physiotherapists' experiences of adopting an eTool in clinical practice: a post-phenomenological investigation. https://pubmed.ncbi.nlm.nih.gov/31635512<br>• Blumenthal, J., Wilkinson, A., & Chignell, M. (2018). Physiotherapists' and Physiotherapy Students' Perspectives on the Use of Mobile or Wearable Technology in Their Practice. http://dx.doi.org/10.3138/ptc.2016-100.e<br>• Ceprnja, D., Clark, T., Young, J., Lee, R., Flynn, K., & Maka, K. (2023). Evaluating experiences, usability and patient satisfaction with telehealth for tertiary outpatient physiotherapy services during COVID-19: A mixed-methods study. *Physiotherapy Theory & Practice*, *39*(9). https://doi.org/https://dx.doi.org/10.1080/09593985.2022.2059423<br>• Cottrell, M. A., & Russell, T. G. (2020). Telehealth for musculoskeletal physiotherapy. https://pubmed.ncbi.nlm.nih.gov/32560876<br>• Dawson, R., Gilchrist, H., Pinheiro, M., Nelson, K., Bowes, N., Sherrington, C., & Haynes, A. (2024). Experiences of Older Adults, Physiotherapists, and Aged Care Staff in the TOP UP Telephysiotherapy Program: Interview Study of the TOP UP Interventions. *JMIR Aging*, *7*. https://doi.org/https://dx.doi.org/10.2196/53010<br>• Dostie, R., Dunn, H., Marks, W. N., Camden, C., & Lovo, S. (2024). Use of telehealth for paediatric rehabilitation needs of Indigenous children - a scoping review. In.<br>• Duarte-García, D. A., Joya-Velasco, P. V., & Niño-Ramírez, M. J. (2023). *Diseño de una Herramienta Digital Para el Tamizaje de Lesión de Hombro Durante el Desarrollo de Competencias Profesionales en Fisioterapia ; Design of a Digital Tool for Shoulder Injury Screening During the Development of Professional Competencies in Physiotherapy* https://repositorio.udes.edu.co/handle/001/9728<br>• Dunphy, E., Hamilton, F. L., Spasic, I., & Button, K. (2017). Acceptability of a digital health intervention alongside physiotherapy to support patients following anterior cruciate ligament reconstruction. *BMC Musculoskeletal Disorders*, *18*(1). https://doi.org/https://dx.doi.org/10.1186/s12891-017-1846-0<br>• Gaveikaite, V., Grundstrom, C., Lourida, K., Winter, S., Priori, R., Chouvarda, I., & Maglaveras, N. (2020). Developing a strategic understanding of telehealth service adoption for COPD care management: A causal loop analysis of healthcare professionals. *PLoS ONE [Electronic Resource]*, *15*(3). https://doi.org/https://dx.doi.org/10.1371/journal.pone.0229619<br>• Gawronska, A., Zurek-Biesiada, D., Rozkocha, S., Golec, J., Czechowska, D., & Maslon, A. (2017). Two Years of Experience in Implementation of the mobiREH Remote Rehabilitation System Supporting Patients and Physiotherapists. https://journals.ukzn.ac.za/index.php/JISfTeH/article/view/276

- Granviken, F., Meisingset, I., Vasseljen, O., Bach, K., Bones, A. F., & Klevanger, N. E. (2023). Acceptance and use of a clinical decision support system in musculoskeletal pain disorders - the SupportPrim project. *BMC Medical Informatics & Decision Making*, *23*(1). https://doi.org/https://dx.doi.org/10.1186/s12911-023-02399-7
- Hall, J. B., Luechtefeld, J. T., & Woods, M. L. (2021). Adoption of Telehealth by Pediatric Physical Therapists During COVID-19: A Survey Study. *Pediatric Physical Therapy*, *33*(4). https://doi.org/https://dx.doi.org/10.1097/PEP.0000000000000817
- Hall, J. B., Luechtefeld, J. T., & Woods, M. L. (2022). Pediatric Physical Therapists' Perceptions of Telehealth Continuation Post-pandemic: A Thematic Analysis of Open-Ended Survey Responses. *Pediatric Physical Therapy*, *34*(3). https://doi.org/https://dx.doi.org/10.1097/PEP.0000000000000908
- Harel, Y., Romano, A., & Lotan, M. (2024). Remote Physiotherapy for Children with ASD during the COVID-19 Pandemic: A Thematic Analysis of Physical Therapists' Perspectives. https://pubmed.ncbi.nlm.nih.gov/38541836
- Hart, P., Bierwirth, R., Fulk, G., & Sazonov, E. (2014). The design and evaluation of an activity monitoring user interface for people with stroke. *Annual International Conference Of The IEEE Engineering In Medicine And Biology Society*, *2014*. https://doi.org/https://dx.doi.org/10.1109/EMBC.2014.6944973
- Hinman, R. S., Nelligan, R. K., Bennell, K. L., & Delany, C. (2017). "Sounds a Bit Crazy, But It Was Almost More Personal:" A Qualitative Study of Patient and Clinician Experiences of Physical Therapist-Prescribed Exercise For Knee Osteoarthritis Via Skype. *Arthritis Care Res (Hoboken)*, *69*(12), 1834-1844. https://doi.org/10.1002/acr.23218
- Horsley, S., Schock, G., Grona, S. L., Montieth, K., Mowat, B., Stasiuk, K., Boden, C., & Bath, B. (2020). Use of real-time videoconferencing to deliver physical therapy services: A scoping review of published and emerging evidence. *Journal of Telemedicine & Telecare*, *26*(10). https://doi.org/https://dx.doi.org/10.1177/1357633X19854647
- Jesús Martínez de la, C., Manuel, F.-S., Guillermo Adolfo, M.-P., Deirdre, A. H., Adelaida María, C.-S., & Inmaculada Carmen, L.-P. (2021). Physical Therapists' Opinion of E-Health Treatment of Chronic Low Back Pain. https://doi.org/10.3390/ijerph18041889
- Lawford, B. J., Bennell, K. L., Kasza, J., & Hinman, R. S. (2018). Physical Therapists' Perceptions of Telephone- and Internet Video-Mediated Service Models for Exercise Management of People With Osteoarthritis. *Arthritis Care Res (Hoboken)*, *70*(3), 398-408. https://doi.org/10.1002/acr.23260
- Lawford, B. J., Delany, C., Bennell, K. L., & Hinman, R. S. (2019). "I Was Really Pleasantly Surprised": Firsthand Experience and Shifts in Physical Therapist Perceptions of
- Telephone-Delivered Exercise Therapy for Knee Osteoarthritis-A Qualitative Study. *Arthritis care & research*, *71*(4). https://doi.org/https://dx.doi.org/10.1002/acr.23618
- Lee, A. C., Davenport, T. E., & Randall, K. (2018). Telehealth Physical Therapy in Musculoskeletal Practice. *Journal of Orthopaedic & Sports Physical Therapy*, *48*(10). https://doi.org/https://dx.doi.org/10.2519/jospt.2018.0613
- Martin, R., Mandrusiak, A., Russell, T., & Forbes, R. (2022). New-graduate physiotherapists' training needs and readiness for telehealth. *Physiotherapy Theory & Practice*, *38*(13). https://doi.org/https://dx.doi.org/10.1080/09593985.2021.1955423
- Maurice, M. (2011). Telerehabilitation In South Africa – Is There A Way Forward? https://doi.org/10.5195/ijt.2011.6069
- Michell, A., Besomi, M., Seron, P., Voigt, M., Cubillos, R., Parada, F., Urrejola, O., Barbosa, T., De Oliveira, D., Bianca, J., Moreno, J., Pinzón, I., Aguirre, C., Hinman, R., Bennell, K., & Russell, T. (2022). Implementation of physiotherapy telerehabilitation before and post Covid-19 outbreak: A comparative narrative between South American countries and Australia. https://repositorio.udd.cl/handle/11447/7404

- Minghelli, B., Soares, A., Guerreiro, A., Ribeiro, A., Cabrita, C., Vitoria, C., Nunes, C., Martins, C., Gomes, D., Goulart, F., Dos Santos, R. M., & Antunes, R. (2020). Physiotherapy services in the face of a pandemic. *Revista da Associacao Medica Brasileira*, *66(4)*. https://doi.org/https://dx.doi.org/10.1590/1806-9282.66.4.491
- Narváez, F., Marín-Castrillón, D. M., Cuenca, M. C., & Latta, M. A. (2017). Development and implementation of technologies for physical telerehabilitation in Latin America: ; Desarrollo e implementación de tecnologías para telerehabilitación física en América Latina: una revisión sistemática de literatura, programas y proyectos. https://revistas.itm.edu.co/index.php/tecnologicas/article/view/721
- Negrini, S., Donzelli, S., Negrini, A., Negrini, A., Romano, M., & Zaina, F. (2020). Feasibility and Acceptability of Telemedicine to Substitute Outpatient Rehabilitation Services in the COVID-19 Emergency in Italy: An Observational Everyday Clinical-Life Study. *Archives of Physical Medicine & Rehabilitation*, *101*(11). https://doi.org/https://dx.doi.org/10.1016/j.apmr.2020.08.001
- Nguyen, T. (2015). *Implementace mHealth do oblasti fyzioterapie ; Implementation of mHealth into Physiotherapy* https://vskp.vse.cz/eid/45545
- Noblin, A., Shettian, M., Cortelyou-Ward, K., & Schack Dugre, J. (2017). Exploring physical therapists' perceptions of mobile application usage utilizing the FITT framework. *Informatics for health & social care*, *42*(2). https://doi.org/https://dx.doi.org/10.1080/17538157.2016.1178118
- Odole, A. C., Afolabi, K. O., Ushie, B. A., & Odunaiya, N. A. (2020). Views of physiotherapists from a low resource setting about physiotherapy at a distance: a qualitative study. *European Journal of Physiotherapy*, *22(1)*. https://doi.org/https://dx.doi.org/10.1080/21679169.2018.1549272
- Oi-Mean, F., Jing-Mei, Y., Suziah, S., & Dayang Rohaya Awang, R. (2014). MOBILE HEALTH AWARENESS IN PRE-DETECTION OF MILD STROKE SYMPTOMS. https://doi.org/10.3844/jcssp.2014.2383.2394
- Pacheco, T. B. F., Bezerra, D. A., de S Silva, J. P., Cacho, Ê. W. A., de Souza, C. G., & Cacho, R. O. (2021). The Implementation of Teleconsultations in a Physiotherapy Service During Covid-19 Pandemic in Brazil: a Case Report. https://pubmed.ncbi.nlm.nih.gov/34345343
- Palyo, S. A., Schopmeyer, K. A., & McQuaid, J. R. (2012). Tele-pain management: use of videoconferencing technology in the delivery of an integrated cognitive-behavioral and physical therapy group intervention. *Psychological Services*, *9*(2). https://doi.org/https://dx.doi.org/10.1037/a0025987
- Partanen, T., Seppanen-Jarvela, R., Hiekkala, S., & Lindh, J. (2023). Telerehabilitation in the Finnish Outpatient Rehabilitation Setting from the Perspective of the Socio-Technical Systems Theory. *International Journal of Environmental Research & Public Health [Electronic Resource]*, *20*(15). https://doi.org/https://dx.doi.org/10.3390/ijerph20156519
- Perron, A. E., Garg, H., Gallagher, S., Kennedy, B., Oxborough, S., Schultz, E., Thielman, G., & Zhang, Q. (2023). Addressing Opportunities and Barriers in Telehealth Neurologic Physical Therapy: Strategies to Advance Practice. *Journal of Neurologic Physical Therapy*, *47*(4). https://doi.org/https://dx.doi.org/10.1097/NPT.0000000000000454
- Raimer, M. (2023). Analyse, Design und Entwicklung einer Serious Games Plattform zur Unterstützung der Telerehabilitation nach einer Knöchelverletzung. In.
- Ramachandran, H. J., Oh, J. L., Cheong, Y. K., Jiang, Y., Teo, J. Y. C., Seah, C. W. A., Yu, M., & Wang, W. (2023). Barriers and facilitators to the adoption of digital health interventions for COPD management: A scoping review. *Heart and Lung*, *59*. https://doi.org/https://dx.doi.org/10.1016/j.hrtlng.2023.02.004
- Roitenberg, N., Pincus, T., & Ben Ami, N. (2022). Physiotherapy services during the COVID-19 pandemic: A mediated model of physiotherapists' self-efficacy, tele-physiotherapy role stressors, and motivation to provide tele-physiotherapy.

Physiotherapy theory and practice. https://doi.org/https://dx.doi.org/10.1080/09593985.2022.2138662

- Ross, M. H., Russell, T., Bennell, K. L., Campbell, P. K., Kimp, A. J., Foster, N. E., & Hinman, R. S. (2023). Technical issues occur but are infrequent and have little impact on physiotherapist-delivered videoconferencing consultations for knee osteoarthritis: A descriptive study. https://pubmed.ncbi.nlm.nih.gov/37269590
- Rothgangel, A., Kanera, I., van den Heuvel, R., Wientgen, M., Jamin, G., Lenssen, T., & Braun, S. (2022). Physiotherapists' clinical use and acceptance of a telemonitoring platform during anterior cruciate ligament rehabilitation:a prospective clinical study. https://cris.maastrichtuniversity.nl/en/publications/1c231b28-ef55-49db-a299-3e80512ae52f
- Sanchez-Rodriguez, M. T., Pinzon-Bernal, M. Y., Jimenez-Antona, C., Laguarta-Val, S., Sanchez-Herrera-Baeza, P., Fernandez-Gonzalez, P., & Cano-de-la-Cuerda, R. (2023). Designing an Informative App for Neurorehabilitation: A Feasibility and Satisfaction Study by Physiotherapists. *Healthcare*, *11*(18). https://doi.org/https://dx.doi.org/10.3390/healthcare11182549
- Simeoni, R., Colonnelli, F., Eutizi, V., Marchetti, M., Paolini, E., Papalini, V., Punturo, A., Salvo, A., Scipinotti, N., Serpente, C., Barbini, E., Troscia, R., Maccioni, G., & Giansanti, D. (2021). The Social Robot and the Digital Physiotherapist: Are We Ready for the Team Play? *Healthcare*, *9*(11). https://doi.org/https://dx.doi.org/10.3390/healthcare9111454
- Spasic, I., Button, K., Divoli, A., Gupta, S., Pataky, T., Pizzocaro, D., Preece, A., van Deursen, R., & Wilson, C. (2015). TRAK App Suite: A Web-Based Intervention for Delivering Standard Care for the Rehabilitation of Knee Conditions. *JMIR Research Protocols*, *4*(4). https://doi.org/https://dx.doi.org/10.2196/resprot.4091
- van Tilburg, M. L., Kloek, C. J. J., Foster, N. E., Ostelo, R., Veenhof, C., Staal, J. B., & Pisters, M. F. (2023). Development and feasibility of stratified primary care physiotherapy integrated with eHealth in patients with neck and/or shoulder complaints: results of a mixed methods study. *BMC Musculoskeletal Disorders*, *24*(1). https://doi.org/https://dx.doi.org/10.1186/s12891-023-06272-6
- Vorrink, S., Huisman, C., Kort, H., Troosters, T., & Lammers, J.-W. (2017). Perceptions of Patients With Chronic Obstructive Pulmonary Disease and Their Physiotherapists Regarding the Use of an eHealth Intervention. https://lirias.kuleuven.be/handle/123456789/600581
- Weber, F., Kloek, C., Arntz, A., Gruneberg, C., & Veenhof, C. (2022). Blended care in patients with knee and/or hip osteoarthritis in physical therapy: a Delphi study on needs and preconditions. *medRxiv.*, *26*. https://doi.org/https://dx.doi.org/10.1101/2022.10.25.22281495

Not DHI

- Alder, G., Taylor, D., Rashid, U., Olsen, S., Brooks, T., Terry, G., Niazi, I. K., & Signal, N. (2023). A Brain Computer Interface Neuromodulatory Device for Stroke Rehabilitation: Iterative User-Centered Design Approach. *JMIR Rehabilitation And Assistive Technologies*, *10*. https://doi.org/https://dx.doi.org/10.2196/49702
- Morris, L., Diteesawat, R. S., Rahman, N., Turton, A., Cramp, M., & Rossiter, J. (2023). The-state-of-the-art of soft robotics to assist mobility: a review of physiotherapist and patient identified limitations of current lower-limb exoskeletons and the potential soft-robotic solutions. *Journal of Neuroengineering & Rehabilitation*, *20*(1). https://doi.org/https://dx.doi.org/10.1186/s12984-022-01122-3
- Vaughan-Graham, J., Brooks, D., Rose, L., Nejat, G., Pons, J., & Patterson, K. (2020). Exoskeleton use in post-stroke gait rehabilitation: a qualitative study of the perspectives of persons post-stroke and physiotherapists. *Journal of Neuroengineering & Rehabilitation*, *17*(1). https://doi.org/https://dx.doi.org/10.1186/s12984-020-00750-x

- Zabel, S., Lockhart, Z., Badiani, N., Cornish, J., Falzon, L., Flis, A., Patterson, K., Gregor, S., & Vaughan-Graham, J. (2022). Physiotherapy students' perspectives on the use and implementation of exoskeletons as a rehabilitative technology in clinical
- settings. *Disability & Rehabilitation Assistive Technology*, *17*(7). https://doi.org/https://dx.doi.org/10.1080/17483107.2020.1818139
- **Unclear distinction of findings from other populations**
- Ann, S. (2020). Identifying Motives for Implementing eHealth by using Activity Theory. https://doi.org/10.3390/su12041298
- Bairapareddy, K. C., Alaparthi, G. K., Jitendra, R. S., & Prathiksha. (2021). "We are so close; yet too far": perceived barriers to smartphone-based telerehabilitation among healthcare providers and patients with Chronic Obstructive Pulmonary Disease in India. In.
- Boland, M. R., Kruis, A. L., Huygens, S. A., Tsiachristas, A., Assendelft, W. J., Gussekloo, J., Blom, C. M., Chavannes, N. H., & Rutten-van Molken, M. P. (2015). Exploring the variation in implementation of a COPD disease management programme and its impact on health outcomes: a post hoc analysis of the RECODE cluster randomised trial. *NPJ Primary Care Respiratory Medicine*, *25*. https://doi.org/https://dx.doi.org/10.1038/npjpcrm.2015.71
- Brouns, B., Meesters, J. J. L., Wentink, M. M., de Kloet, A. J., Arwert, H. J., Vliet Vlieland, T. P. M., Boyce, L. W., & van Bodegom-Vos, L. (2018). Why the uptake of eRehabilitation programs in stroke care is so difficult-a focus group study in the Netherlands. *Implementation Science*, *13*(1). https://doi.org/https://dx.doi.org/10.1186/s13012-018-0827-5
- Camden, C., & Silva, M. (2021). Pediatric Teleheath: Opportunities Created by the COVID-19 and Suggestions to Sustain Its Use to Support Families of Children with Disabilities. *Physical & Occupational Therapy in Pediatrics*, *41*(1). https://doi.org/https://dx.doi.org/10.1080/01942638.2020.1825032
- Cartledge, S., Rawstorn, J. C., Tran, M., Ryan, P., Howden, E. J., & Jackson, A. (2022). Telehealth is here to stay but not without challenges: a consultation of cardiac rehabilitation clinicians during COVID-19 in Victoria, Australia. *European Journal of Cardiovascular Nursing*, *21*(6). https://doi.org/https://dx.doi.org/10.1093/eurjcn/zvab118
- Casillas, A., Valdovinos, C., Wang, E., Abhat, A., Mendez, C., Gutierrez, G., Portz, J., Brown, A., & Lyles, C. R. (2022). Perspectives from leadership and frontline staff on telehealth transitions in the Los Angeles safety net during the COVID-19 pandemic and beyond. *Frontiers in Digital Health*, *4(no pagination)*, Article 944860. https://doi.org/https://dx.doi.org/10.3389/fdgth.2022.944860
- Curtz, J., Mazariegos, J., Adeyemo, J., Smith, C., DiOrio, A., Logan, K., & Russell, H. (2021). Responding to an Emerging Need: Implementing Telehealth in Acute Hospital Rehabilitation. *Archives of Physical Medicine & Rehabilitation*, *102*(9). https://doi.org/https://dx.doi.org/10.1016/j.apmr.2021.05.006
- Damhus, C. S., Emme, C., & Hansen, H. (2018). Barriers and enablers of COPD telerehabilitation - a frontline staff perspective. *International Journal of Copd*, *13*. https://doi.org/https://dx.doi.org/10.2147/COPD.S167501
- Epalte, K., Grjadovojs, A., & Berzina, G. (2023). Use of the Digital Assistant Vigo in the Home Environment for Stroke Recovery: Focus Group Discussion With Specialists Working in Neurorehabilitation. *JMIR Rehabilitation And Assistive Technologies*, *10*. https://doi.org/https://dx.doi.org/10.2196/44285
- Farzad, M., MacDermid, J., Ferreira, L., Szekeres, M., Cuypers, S., & Shafiee, E. (2023). A description of the barriers, facilitators, and experiences of hand therapists in providing remote (tele) rehabilitation: An interpretive description approach. *Journal of Hand Therapy*, *36(4)*. https://doi.org/https://dx.doi.org/10.1016/j.jht.2023.06.004
- Frey, S., & Kerkemeyer, L. (2022). Acceptance of digital health applications in non-pharmacological therapies in German statutory healthcare system: Results of an

online survey. *Digital Health*, *8*. https://doi.org/https://dx.doi.org/10.1177/20552076221131142

- Gell, N. M., Smith, P. A., & Wingood, M. (2024). Physical Therapist and Patient Perspectives on Mobile Technology to Support Home Exercise Prescription for People With Arthritis: A Qualitative Study. *Cureus*, *16*(3). https://doi.org/https://dx.doi.org/10.7759/cureus.55899
- Grundstein, M. J., Fisher, C., Titmuss, M., & Cioppa-Mosca, J. (2021). The Role of Virtual Physical Therapy in a Post-Pandemic World: Pearls, Pitfalls, Challenges, and Adaptations. *Physical Therapy*, *101*(9). https://doi.org/https://dx.doi.org/10.1093/ptj/pzab145
- Guise, V., & Wiig, S. (2017). Perceptions of telecare training needs in home healthcare services: a focus group study. *BMC Health Services Research*, *17*(1). https://doi.org/https://dx.doi.org/10.1186/s12913-017-2098-2
- Inskip, J. A., Lauscher, H. N., Li, L. C., Dumont, G. A., Garde, A., Ho, K., Hoens, A. M., Road, J. D., Ryerson, C. J., & Camp, P. G. (2018). Patient and health care professional perspectives on using telehealth to deliver pulmonary rehabilitation. *Chronic Respiratory Disease*, *15*(1). https://doi.org/https://dx.doi.org/10.1177/1479972317709643
- Liljeroos, M., & Arkkukangas, M. (2023). Implementation of Telemonitoring in Health Care : Facilitators and Barriers for Using eHealth for Older Adults with Chronic Conditions. http://urn.kb.se/resolve?urn=urn:nbn:se:du-45273
- Lin, D., Papi, E., & McGregor, A. H. (2019). Exploring the clinical context of adopting an instrumented insole: a qualitative study of clinicians' preferences in England. *BMJ Open*, *9*(4). https://doi.org/https://dx.doi.org/10.1136/bmjopen-2018-023656
- Lopera Escobar, A., Tamayo Montoya, V., Carvajal Cartagena, D., Yepes Gómez, K., & Arcos Pedroza, K. Y. (2022). Implementación de un programa de Telerehabilitación en tiempos de aislamiento social 2020. https://dialnet.unirioja.es/servlet/oaiart?codigo=8958788
- Louie, D. R., Bird, M. L., Menon, C., & Eng, J. J. (2020). Perspectives on the prospective development of stroke-specific lower extremity wearable monitoring technology: a qualitative focus group study with physical therapists and individuals with stroke. *Journal of Neuroengineering & Rehabilitation*, *17*(1). https://doi.org/https://dx.doi.org/10.1186/s12984-020-00666-6
- Maddahi, A., Bani Hani, J., Asgari, A., Nassiri, A. M., & Choukou, M. A. (2021). Therapists' perspectives on a new portable hand telerehabilitation platform for home-based personalized treatment of stroke patients. *European Review for Medical & Pharmacological Sciences*, *25*(18). https://doi.org/https://dx.doi.org/10.26355/eurrev_202109_26797
- Malliaras, P., Merolli, M., Williams, C. M., Caneiro, J. P., Haines, T., & Barton, C. (2021). 'It's not hands-on therapy, so it's very limited': Telehealth use and views among allied health clinicians during the coronavirus pandemic. *Musculoskeletal Science & Practice*, *52*. https://doi.org/https://dx.doi.org/10.1016/j.msksp.2021.102340
- Marja, Ä., Jori, R., & Pirjo, K. (2022). eHealth challenges in rehabilitation processes - rehabilitation professionals’ experiences in the North Savo region. https://doi.org/10.23996/fjhw.111720
- Morris, J., Wallace, T., Sheehan, L., Souders, L., Jones, M., DeRuyter, F., & Thompson, N. (2019). Clinician Perspectives on mHealth/mRehab Interventions and Technologies. *Archives of Physical Medicine and Rehabilitation*, *100(10)*. https://doi.org/https://dx.doi.org/10.1016/j.apmr.2019.08.141
- Munce, S., Andreoli, A., Bayley, M., Guo, M., Inness, E. L., Kua, A., & McIntyre, M. (2023). Clinicians' Experiences of Implementing a Telerehabilitation Toolkit During the COVID-19 Pandemic: Qualitative Descriptive Study. *JMIR Rehabilitation And Assistive Technologies*, *10*. https://doi.org/https://dx.doi.org/10.2196/44591

- Nguyen, G., King, K., & Stirling, L. (2023). Telerehabilitation use and experiences in occupational and physical therapy through the early stages of the COVID-19 pandemic. *PLoS ONE [Electronic Resource]*, *18*(11).
- Nyberg, A., Sondell, A., Lundell, S., Marklund, S., Tistad, M., & Wadell, K. (2022). Experiences of using an electronic health (eHealth)-tool among healthcare professionals involved in COPD management - a qualitative analysis. *European Respiratory Journal. Conference: European Respiratory Society International Congress, ERS*, *60*(Supplement 66). https://doi.org/https://dx.doi.org/10.1183/13993003.congress-2022.2983
- Papi, E., Murtagh, G. M., & McGregor, A. H. (2016). Wearable technologies in osteoarthritis: a qualitative study of clinicians' preferences. *BMJ Open*, *6*(1). https://doi.org/https://dx.doi.org/10.1136/bmjopen-2015-009544
- Paul, L., Thomson, K., Asibey, S. O., Brady, M., van Wijck, F., Antwi, D., Opoku, E. N., & Sarfo, F. S. (2024). Views of Service Users, Their Family or Carers, and Health Care Professionals on Telerehabilitation for People With Neurological Conditions in Ghana: Qualitative Study. *JMIR MHealth and UHealth*, *12*. https://doi.org/https://dx.doi.org/10.2196/49501
- Paul, S. S., Hubbard, A., Johnson, J., & Dennis, S. M. (2023). Transition to a virtual model of physiotherapy and exercise physiology in response to COVID-19 for people in a rural Australia: Is it a viable solution to increase access to allied health for rural
- populations? *PLoS ONE*, *18(1 January) (no pagination)*, Article e0280876. https://doi.org/https://dx.doi.org/10.1371/journal.pone.0280876
- Schwarz, M., Coccetti, A., Draheim, M., & Gordon, G. (2020). Perceptions of allied health staff of the implementation of an integrated electronic medical record across regional and metropolitan settings. *Australian Health Review*, *44*(6). https://doi.org/https://dx.doi.org/10.1071/AH19024
- Seebacher, B., Bergmann, E., Geimer, C., Kahraman, T., Reindl, M., & Diermayr, G. (2024). Factors influencing the willingness to adopt telerehabilitation among rehabilitation professionals in Austria and Germany: a survey comparing data before and during COVID-19. *Disability & Rehabilitation*, *46*(6). https://doi.org/https://dx.doi.org/10.1080/09638288.2023.2193428
- Simpson, L. A., Menon, C., Hodgson, A. J., Ben Mortenson, W., & Eng, J. J. (2021). Clinicians' perceptions of a potential wearable device for capturing upper limb activity post-stroke: a qualitative focus group study. *Journal of Neuroengineering & Rehabilitation*, *18*(1). https://doi.org/https://dx.doi.org/10.1186/s12984-021-00927-y
- Sobrepera, M. J., Elfishawy, J., Nguyen, A. T., Prosser, L. P., & Johnson, M. J. (2024). Insights on Telecommunication Use by Rehabilitation Therapists Before, During, and Beyond COVID-19. *Archives of Rehabilitation Research and Clinical Translation*, *(no pagination)*, Article 100326. https://doi.org/https://dx.doi.org/10.1016/j.arrct.2024.100326
- Stinson, J. N., Lalloo, C., Harris, L., Isaac, L., Campbell, F., Brown, S., Ruskin, D., Gordon, A., Galonski, M., Pink, L. R., Buckley, N., Henry, J. L., White, M., & Karim, A. (2014). ICanCope with PainTM: User-centred design of a web- and mobile-based self-management program for youth with chronic pain based on identified health care needs. *Pain Research and Management*, *19(5)*. https://doi.org/http://dx.doi.org/10.1155/2014/935278
- Stock, R., Parviz Gaarden, A., & Langorgen, E. (2023). Stroke survivors' and physiotherapists' opinions and perceptions about the potential of a wearable system designed to facilitate motivation for home exercise. *Neurorehabilitation and Neural Repair*, *37(5)*. https://doi.org/https://dx.doi.org/10.1177/154596832311594
- Swink, L. A., Mealer, M. L., Miller, M. J., Anderson, C. B., Cook, P. F., Stevens-Lapsley, J. E., & Christiansen, C. L. (2024). Telehealth Walking Self-Management for Individuals With Amputation: A Qualitative Study of Therapist Perspectives on

- Adoption. *Physical Therapy*, *104*(2). https://doi.org/https://dx.doi.org/10.1093/ptj/pzad155
- Toft, B. S., Rodkjaer, L. O., Sorensen, L., Saugbjerg, M. R., Bekker, H. L., & Modrau, I. S. (2024). Feasibility of early digital health rehabilitation after cardiac surgery in the elderly: a qualitative study. *BMC Health Services Research*, *24*(1). https://doi.org/https://dx.doi.org/10.1186/s12913-024-10601-3
- Toftdahl, A. K. S., Ibsen, S., Pape-Haugaard, L. B., & Riis, A. (2023). Therapists' experiences with implementing new documentation practices for low back pain in electronic health care records: an interview study. *BMC Research Notes*, *16*(1). https://doi.org/https://dx.doi.org/10.1186/s13104-023-06567-w
- Wentink, M. M., L, V. A. N. B.-V., Brouns, B., Arwert, H. J., Vlieland, T., AJ, D. E. K., & Meesters, J. J. L. (2018). What is Important in E-health Interventions for Stroke Rehabilitation? A Survey Study among Patients, Informal Caregivers and Health Professionals. *International Journal of Telerehabilitation*, *10*(1). https://doi.org/https://dx.doi.org/10.5195/ijt.2018.6247
- Wilde, L. J., Percy, C., Clark, C., Ward, G., Wark, P. A., & Sewell, L. (2023). Views and experiences of healthcare practitioners supporting people with COPD who have used activity monitors: "More than just steps". *Respiratory Medicine*, *218*. https://doi.org/https://dx.doi.org/10.1016/j.rmed.2023.107395
- Willcox, J. C., van der Pligt, P., Ball, K., Wilkinson, S. A., Lappas, M., McCarthy, E. A., & Campbell, K. J. (2015). Views of Women and Health Professionals on mHealth Lifestyle Interventions in Pregnancy: A Qualitative Investigation. *JMIR MHealth and UHealth*, *3*(4). https://doi.org/https://dx.doi.org/10.2196/mhealth.4869
- Wittmeier, K. D. M., Hammond, E., Tymko, K., Burnham, K., Janssen, T., Pablo, A. J., Russell, K., Pierce, S., Costello, C., & Protudjer, J. L. P. (2022). "Another Tool in Your Toolkit": Pediatric Occupational and Physical Therapists' Perspectives of Initiating Telehealth during the COVID-19 Pandemic. *Physical & Occupational Therapy in Pediatrics*, *42*(5). https://doi.org/https://dx.doi.org/10.1080/01942638.2022.2065898
- Wu, F., Burt, J., Chowdhury, T., Fitzpatrick, R., Martin, G., van der Scheer, J. W., & Hurst, J. R. (2021). Specialty COPD care during COVID-19: patient and clinician
- perspectives on remote delivery. *BMJ open respiratory research*, *8*(1). https://doi.org/https://dx.doi.org/10.1136/bmjresp-2020-000817
- Yung, A. (2017). Adoption of Electronic Health Record System in Community-Based Physiotherapy Clinics: A Pilot Case Study. *Studies in Health Technology & Informatics*, *234*. https://ovidsp.ovid.com/ovidweb.cgi?T=JS&CSC=Y&NEWS=N&PAGE=fulltext&D=med14&AN=28186074
- **Full text missing**
- Author not stated 2018 https://eresearch.qmu.ac.uk/handle/20.500.12289/9445
- Helle, N. (2018). Adoption of Mobile Applications in Physiotherapy : role of influencing factors in physiotherapists' adoption decision. In.
- Simpson, L. A., Eng, J. J., Sadarangani, G., Hodgson, A. J., & Menon, C. (2015). Wearable devices to capture upper limb activity post stroke: Clinician perceptions of their utility. *International Journal of Stroke*, *4)*. https://doi.org/https://dx.doi.org/10.1111/ijs.12633-2
- Sivarajah, L., Bayley, M., MacKay-Lyons, M., Brooks, D., Howe, J. A., McDonald, A., Mihailidis, A., Solomon, P., Kel-Loway, L., & Salbach, N. (2017). Usage and perceptions of iWalk-A mobile application designed to facilitate physical therapists uptake of the 10-metre and 6-minute walk test post stroke. *Cerebrovascular Diseases*, *43(Supplement 1)*. https://ovidsp.ovid.com/ovidweb.cgi?T=JS&CSC=Y&NEWS=N&PAGE=fulltext&D=emed18&AN=619777453
- **Not able to retrieve**
- O'Neil, J., van Ierssel, J., King, J., & Sveistrup, H. (2023). Telerehabilitation

- Implementation: Perspectives from Physiotherapists Working in Complex Care. http://dx.doi.org/10.3138/ptc-2022-0072
- **Not PT delivery setting**
- Davies, L., Lawford, B., Bennell, K. L., Russell, T., & Hinman, R. S. (2023). ´
- Telehealth education and training in entry-to-practice physiotherapy programs in Australian universities: A qualitative study with university educators. *Musculoskeletal Care*, *21*(2). https://doi.org/https://dx.doi.org/10.1002/msc.1723
- Davies, L., Lawford, B., Bennell, K. L., Russell, T., & Hinman, R. S. (2023). Telehealth education and training in entry-to-practice physiotherapy programs in Australian universities: A qualitative study with university educators. *Musculoskeletal Care*, *21*(2). https://doi.org/https://dx.doi.org/10.1002/msc.1723
- Deutsch, J. E., Palmieri, J. L., Gorin, H., Wendell, A., Wohn, D. Y., & Damodaran, H. (2023). Student and Faculty Perspectives on the Usefulness and Usability of a Digital Health Educational Tool to Teach Standardized Assessment of Persons After Stroke: Mixed Methods Study. *JMIR medical education*, *9*. https://doi.org/https://dx.doi.org/10.2196/44361
- Johnson, S. G., Titlestad, K. B., Larun, L., Ciliska, D., & Olsen, N. R. (2021). Experiences with using a mobile application for learning evidence-based practice in health and social care education: An interpretive descriptive study. https://hdl.handle.net/11250/2770123
- Wentink, M. M., Siemonsma, P. C., van Bodegom-Vos, L., de Kloet, A. J.,
- Verhoef, J., Vlieland, T., & Meesters, J. J. L. (2019). Teachers' and students' perceptions on barriers and facilitators for eHealth education in the curriculum of functional exercise and physical therapy: a focus groups study. *BMC Medical Education*, *19*(1). https://doi.org/https://dx.doi.org/10.1186/s12909-019-1778-5

Abstract/Poster

- Aartolahti, E., Oduor, M., Tumusiime, D., & Korniloff, K. (2024). Rwandan Physiotherapists Experiences of a Digital Rehabilitation Solution: A Pilot Usability Study. *Archives of Physical Medicine and Rehabilitation*, *105(4)*. https://doi.org/https://dx.doi.org/10.1016/j.apmr.2024.02.583
- Aljahdali, S., Latchem-Hastings, J., Hamana, K., & Button, K. (2022). Exploring the Perception of End-Users in Introducing a Supported Self-Management Web-Based Intervention for Knee Conditions (Trak) in Saudi Arabia: A Qualitative Study.
- *Osteoarthritis and Cartilage*, *30(Supplement 1)*. https://doi.org/https://dx.doi.org/10.1016/j.joca.2022.02.521
- Bhardwaj, A., Hayes, P., Browne, J., Grealis, S., Maguire, D., O'Hora, J., Dowling, I., Kennedy, N., & Toomey, C. (2023). Barriers and Enablers to Uptake of Evidence-Based Online Exercise Treatments for Osteoarthritis. *Annals of the Rheumatic Diseases*, *82(Supplement 1)*. https://doi.org/https://dx.doi.org/10.1136/annrheumdis-2023-eular.1228
- Bijker, L., Scholten-Peeters, G. G. M., Busink, V., Cuijpers, P., Donker, M., De Wit, L. M., & Coppieters, M. W. (2022). The usability of a blended biopsychosocial treatment 'Back2Action' for patients with persistent spinal pain from the perspective of physiotherapists. *Pain Practice*, *22(Supplement 2)*. https://doi.org/https://dx.doi.org/10.1111/papr.13128
- Browne, S., Boardman, R., Arthurs, N., O'Donnell, S., Doyle, G., Kechadi, T., Case, L., Tully, L., & O'Malley, G. (2020). A clinical portal for childhood obesity management: Acceptability and usability among health care professionals. *Obesity Reviews. Conference: European and International Congress on Obesity, ECOICO*, *21*(SUPPL 1). https://doi.org/https://dx.doi.org/10.1111/obr.13118
- Chugh, D., Waite, G., Harniess, P., & Alderson, L. (2019). Facilitators and barriers to real-world adoption of video exercise gaming technology in the physiotherapy department at Great Ormond Street Hospital. *Archives of Disease in Childhood*,

104(Supplement 4). https://doi.org/https://dx.doi.org/10.1136/archdischild-2019-gosh.70

- Cochrane, K. (2019). Electronic health records and the standard of note writing in community Physiotherapy. *Physiotherapy (United Kingdom)*, *105(Supplement 1)*. https://doi.org/https://dx.doi.org/10.1016/j.physio.2018.11.133
- Coronado, R., Block, S., Gonzalez, K., Rhoten, B., Brintz, C., McKernan, L., Kirkhart, T.,
- Wegener, S., & Archer, K. (2022). Patient and physical therapist experiences with integrating an eHealth pain self-management program into clinical care. *Journal of Clinical and Translational Science*, *6(Supplement 1)*. https://doi.org/https://dx.doi.org/10.1017/cts.2022.306
- Corriveau, H., Tousignant, M., Gaboury, I., & Page, C. (2019). Improvement of rehabilitation services-telerehabilitation: Understanding the constraints and
- facilitators to better defeat clinicians' reticence. *European Stroke Journal*, *4(Supplement 1)*. https://doi.org/https://dx.doi.org/10.1177/2396987319845581
- Cottrell, M., Hill, A., O'Leary, S., Raymer, M., & Russell, T. (2016). Telerehabilitation as an additional service delivery option within an Australian orthopaedic physiotherapy screening service: A needs assessment. *Manual Therapy*, *Conference: IFOMPT 2016 Conference. Glasgow United Kingdom. 25*. https://doi.org/https://dx.doi.org/10.1016/j.math.2016.05.045
- Fowler King, B., MacDonald, J., Stoff, L., Nettnin, E., Jayaraman, A., Goldman, J. G., & Rafferty, M. (2023). Activity Monitoring in Parkinson Disease: A Qualitative Study of Implementation Determinants. *Journal of Neurologic Physical Therapy*, *47*(4). https://doi.org/https://dx.doi.org/10.1097/NPT.0000000000000451
- French, E. H., Reinikka, K. J., & Huijbregts, M. P. (2010). Tele-rehab: The feasibility of using telemedicine to deliver interprofessional stroke rehabilitation consultations in northern, rural and remote communities. *Stroke*, *41(7)*. https://doi.org/https://dx.doi.org/10.1161/STR.0b013e3181e6c862
- Grande, S. W., Kotzbauer, G., Hager, A., Martensson, M., & Longacre, M. (2017). Improving care for pediatric cystic fibrosis in Sweden using a successful mHealth patient support system. *Pediatric Pulmonology*, *52(Supplement 47)*. https://doi.org/https://dx.doi.org/10.1002/ppul.23840
- Kaur, A., Wadhwa, D., Thakur, R., & Mande, M. (2023). Physiotherapist Knowledge, Beliefs and Barrier Regarding Using Artificial Intelligence in Prevention and Rehabilitation of Falls: A Cross-Sectional Study. *Aging Clinical and Experimental Research*, *35(Supplement 1)*. https://doi.org/https://dx.doi.org/10.1007/s40520-023-02442-7
- Keel, S., Schmid, A., & Keller, F. (2023). Digital technology in physiotherapy consultations ::problem-solving sequences and recruitments. In.
- Macdonald, G., Leese, J., Backman, C., Davis, A., Townsend, A. F., Avina-Zubieta, J. A., Gromala, D., & Li, L. (2015). Integrating wearable physical activity monitoring tools into rehabilitation practice for patients with arthritis: The healthcare professional perspective. *Arthritis and Rheumatology. Conference: American College of Rheumatology/Association of Rheumatology Health Professionals Annual Scientific*
- *Meeting, ACR/ARHP*, *67*(SUPPL. 10). https://doi.org/https://dx.doi.org/10.1002/art.39448
- Morris, J., Jones, M., Thompson, N., Wallace, T., & Deruyter, F. (2019). Clinician perspectives on mrehab interventions and technologies for people with disabilities in the united states: A national survey. *International Journal of Environmental Research and Public Health*, *16(21) (no pagination)*, Article 4220. https://doi.org/https://dx.doi.org/10.3390/ijerph16214220
- Nicholas, K. A., Al-Amri, M., Davies, J. L., Sparkes, V., & Button, K. (2019). A qualitative

- evaluation of physiotherapists acceptability of a clinical sensor based approach to movement feedback rehabilitation. *Osteoarthritis and Cartilage*, *27(Supplement 1)*. https://doi.org/https://dx.doi.org/10.1016/j.joca.2019.02.483
- Revenas, A. (2016). Co-designing a mobile internet service for self-management of physical activity in rheumatoid arthritis. *Annals of the Rheumatic Diseases*, *75(Supplement 2)*. https://doi.org/https://dx.doi.org/10.1136/annrheumdis-2016-eular.6208
- Revenas, A., Opava, C. H., Demmelmaier, I., Keller, C., & Asenlof, P. (2014). Development of a Web and Mobile Application (WeMApp) to support physical activity in rheumatoid arthritis: Results from the second step of a co-design process. *Scandinavian Journal of Rheumatology*, *127)*. https://doi.org/https://dx.doi.org/10.3109/03009742.2014.946235
- Roseen, E. J., Coash, E., Laird, L., Zhang, W., Mittman, B., Reed, E., Bravatti, M. L., Trinquart, L., Lavretsky, H., Saper, R., & Wang, C. (2024). Barriers and Facilitators to Use Remote Tai Chi for Knee Osteoarthritis in Large Healthcare Systems: A Qualitative Study. *Osteoarthritis and Cartilage*, *32(Supplement 1)*. https://doi.org/https://dx.doi.org/10.1016/j.joca.2024.02.852
- Sidhanee, A., Makupe, P., & Delano, P. (2021). Paediatric Oncology Rehabilitation during the Covid-19 Pandemic: The Experience of 4 Centres Globally. *Pediatric Blood and Cancer. Conference: 53rd Annual Congress of the International Society of Paediatric Oncology, SIOP*, *68*(SUPPL 5). https://doi.org/https://dx.doi.org/10.1002/pbc.29349
- Simmons, R., Lempp, H., Galloway, J., Oldrieve, H., & Bearne, L. M. (2019). Physical activity maintenance and digital health interventions in people with rheumatoid
- arthritis: a qualitative study. *Physiotherapy (United Kingdom)*, *105(Supplement 1)*. https://doi.org/https://dx.doi.org/10.1016/j.physio.2018.11.263
- Timmer, M., Kloek, C., De Kleijn, P., Schutgens, R., Veenhof, C., & Pisters, M. (2019). E-exercise haemophilic arthropathy: Development of a blended physiotherapy intervention. *Haemophilia*, *25(Supplement 1)*. https://doi.org/https://dx.doi.org/10.1111/hae.13666
- Tonga, E., Srikesavan, C., Williamson, E., & Lamb, S. (2020). A hand exercises mhealth app for patients with rheumatoid arthritis: development, design and usability study in turkey. *Annals of the Rheumatic Diseases*, *79(SUPPL 1)*. https://doi.org/https://dx.doi.org/10.1136/annrheumdis-2020-eular.5496
- Tousignant, M., Moffet, H., Boissy, P., Corriveau, H., Cabana, F., & Marquis, E. (2011). Patients and physiotherapists satisfaction of in-home telerehabilittion for post-knee arthroplasty. *Physiotherapy (United Kingdom)*, *1)*. https://doi.org/https://dx.doi.org/10.1016/j.physio.2011.04.002
- Ullah, S., Maghazil, A., Qureshi, A. Z., Tantawy, S., Moukais, I., & Aldajani, A. (2019). Tele rehabilitation services in Saudi Arabia: Rehabilitation clinicians perspective. *PM and R*, *11(Supplement 2)*. https://doi.org/https://dx.doi.org/10.1002/pmrj.12271
- Wessels, N., Ruiter, E., Hulshof, L., Loohuis, A., Van Gemert-Pijnen, J., Metting, E., Blanker, M., & Van der Worp, H. (2020). Care provider views on app-based treatment for female urinary incontinence: a mixedmethods study. *British Journal of General Practice. Conference: British Journal of General Practice Research Conference, BJGP*, *70*(Supplement 1). https://doi.org/https://dx.doi.org/10.3399/bjgp23X734265
- Wilde, L. J., Clark, C., Percy, C., Ward, G., Wark, P. A., & Sewell, L. (2022). 'It's Definitely the Future': Healthcare Practitioners' Views and Experiences of Activity Monitors to Support People with Chronic Obstructive Pulmonary Disease (Copd). *Thorax*, *77(Supplement 1)*. https://doi.org/https://dx.doi.org/10.1136/thorax-2022-BTSabstracts.307

Duplicates

- Albahrouh, S., & Buabbas, A. (2021). Physiotherapists' perceptions of and willingness to use telerehabilitation in Kuwait during the COVID-19 pandemic. *BMC Medical Informatics and Decision Making*, *21*. https://doi.org/10.1186/s12911-021-01478-x
- D'Souza, A. F., & Rebello, S. R. (2021). Perceptions and Willingness of Physiotherapists in India to Use Telerehabilitation During the COVID-19 Pandemic. *Int J Telerehabil*, *13*(2), e6425. https://doi.org/10.5195/ijt.2021.6425
- Lee, A. C., Deutsch, J. E., Holdsworth, L., Kaplan, S. L., Kosakowski, H., Latz, R.,
- McNeary, L. L., O'Neil, J., Ronzio, O., Sanders, K., Sigmund-Gaines, M., Wiley, M., & Russell, T. (2024). Telerehabilitation in Physical Therapist Practice: A Clinical Practice Guideline from the American Physical Therapy Association. https://pubmed.ncbi.nlm.nih.gov/38513257
- Martínez de la Cal, J., Fernández-Sánchez, M., Matarán-Peñarrocha, G. A., & Hurley, D. (2022). Physical Therapists' Opinion of E-Health Treatment of Chronic Low Back Pain. http://hdl.handle.net/10197/13077
- Tao, L., Carboni-Jimenez, A., Turner, K., Ostbo, N., Aguila, K., Boruff, J., Carrier, M. E., Krishnan, A., Azar, C., Guindon, A., Viens, N., Ahmed, S., Thombs, B. D., & Kwakkenbos, L. (2022). Perceived Barriers and Facilitators of Using Synchronous Telerehabilitation of Physical and Occupational Therapy in Musculoskeletal Disorders: A Scoping Review. *medRxiv.*, *22*. https://doi.org/https://dx.doi.org/10.1101/2022.07.21.22277858

Older than 2014

- Bossen, C., Jensen, L. G., & Udsen, F. W. (2013). Evaluation of a comprehensive EHR based on the DeLone and McLean model for IS success: approach, results, and success factors. *International Journal of Medical Informatics*, *82*(10). https://doi.org/https://dx.doi.org/10.1016/j.ijmedinf.2013.05.010
- Ogasawara, K., Ito, K., Jiang, G., Endoh, A., Sakurai, T., Sato, H., Okuhara, Y., Adachi, T., & Hori, K. (2003). Preliminary clinical evaluation of a video transmission system for home visits. *Journal of Telemedicine & Telecare*, *9*(5). https://doi.org/https://dx.doi.org/10.1258/135763303769211328
- Russell, T. G., Buttrum, P., Wootton, R., & Jull, G. A. (2004). Rehabilitation after total
- knee replacement via low-bandwidth telemedicine: the patient and therapist experience. *Journal of Telemedicine & Telecare*, *10 Suppl 1.*
- Vuononvirta, T., Timonen, M., Keinanen-Kiukaanniemi, S., Timonen, O., Ylitalo, K., Kanste, O., & Taanila, A. (2011). The compatibility of telehealth with health-care delivery. *Journal of Telemedicine & Telecare*, *17*(4). https://doi.org/https://dx.doi.org/10.1258/jtt.2010.100502
- **DHI not specified/broad description**
- Eriksson, P. G. (2020). *Analysis of Physiotherapists Perceptions for Improvement of Digital Innovation ; Analys av fysioterapeuters uppfattningar för förbättring av digital innovation* http://urn.kb.se/resolve?urn=urn:nbn:se:kth:diva-279129
- Estel, K., Scherer, J., Dahl, H., Wolber, E., Forsat, N. D., & Back, D. A. (2022). Potential
- of digitalization within physiotherapy: a comparative survey. *BMC Health Services Research*, *22*(1). https://doi.org/https://dx.doi.org/10.1186/s12913-022-07931-5
- Huynh, T., Kroh, J., & Schultz, C. (2023). Overcoming the not-invented-here syndrome in healthcare: The case of German ambulatory physiotherapists' adoption of digital health innovations. *PLoS ONE [Electronic Resource]*, *18*(12). https://doi.org/https://dx.doi.org/10.1371/journal.pone.0293550
